# Nicotinamide-N-methyltransferase controls behavior, neurodegeneration and lifespan by regulating neuronal autophagy

**DOI:** 10.1371/journal.pgen.1007561

**Published:** 2018-09-07

**Authors:** Kathrin Schmeisser, J. Alex Parker

**Affiliations:** Research Center of the Centre Hospitalier de l‘Université de Montréal (CRCHUM), Department of Neuroscience, Université de Montréal, Quebec, Canada; ETH, SWITZERLAND

## Abstract

Nicotinamide N-methyl-transferase (NNMT) is an essential contributor to various metabolic and epigenetic processes, including the regulating of aging, cellular stress response, and body weight gain. Epidemiological studies show that NNMT is a risk factor for psychiatric diseases like schizophrenia and neurodegeneration, especially Parkinson’s disease (PD), but its neuronal mechanisms of action remain obscure. Here, we describe the role of neuronal NNMT using *C*. *elegans*. We discovered that ANMT-1, the nematode NNMT ortholog, competes with the methyltransferase LCMT-1 for methyl groups from S—adenosyl methionine. Thereby, it regulates the catalytic capacities of LCMT-1, targeting NPRL-2, a regulator of autophagy. Autophagy is a core cellular, catabolic process for degrading cytoplasmic material, but very little is known about the regulation of autophagy during aging. We report an important role for NNMT in regulation of autophagy during aging, where high neuronal ANMT-1 activity induces autophagy via NPRL-2, which maintains neuronal function in old wild type animals and various disease models, also affecting longevity. In younger animals, however, ANMT-1 activity disturbs neuronal homeostasis and dopamine signaling, causing abnormal behavior. In summary, we provide fundamental insights into neuronal NNMT/ANMT-1 as pivotal regulator of behavior, neurodegeneration, and lifespan by controlling neuronal autophagy, potentially influencing PD and schizophrenia risk in humans.

## Author summary

Neuronal disorders are a threat to human health and understanding the underlying genetic and cellular regulatory networks is a first step towards successful treatment. In this context, it has been suggested in epidemiological studies that the metabolism of nicotinamide, specifically the enzyme nicotinamide N-methyl-transferase (NNMT), could contribute to behavioral and neurodegenerative diseases such as Parkinson’s disease (PD) and schizophrenia. We used the simple nematode worm *C*. *elegans* and expressed NNMT in its dopaminergic neurons as this specific neuronal population is primarily affected by both PD and schizophrenia. We found that neuronal NNMT expression in *C*. *elegans* influences behavior, neurodegeneration, and life expectancy. NNMT activity controls the concentration of S—adenosyl methionine (SAM) in the neuronal cell. High NNMT activity leads to low SAM levels that the cell interprets as starvation signal, therefore inducing autophagy, a catabolic process important for balancing energy sources and cell homeostasis. Depending on the age of the worms, this can result in disturbed behavioral paradigms in young animals, or preserves neuronal health and decreases neurodegeneration processes in old individuals. Taken together, these identified mechanisms of NNMT action in *C*. *elegans* neurons could provide novel insights into the development of neuronal disorders in humans.

## Introduction

Neurodegenerative disorders are a major health concern in all aging populations. The modifications associated with these diseases are mostly progressive and irreversible, and while several compounds have been developed that relieve symptoms in the short term, no cure has been identified for any of these conditions. Causes leading to neurodegeneration are both diverse and complex, and include various genetic, epigenetic, and environmental factors.

Parkinson’s disease (PD) is among the most prevalent neurodegenerative diseases and numbers increase steadily with age, reaching approximately 2% of all octogenarians affected worldwide. PD is characterized by dopaminergic (DA) cell death in the substantia nigra in the midbrain, leading to a variety of motor and psychiatric symptoms, such as tremor, depression, and dementia. For decades, PD was considered mostly an idiopathic and sporadic disease with only a small genetic component [1, 2]. However, genetic studies in the last decade have been instrumental in identifying the heredity basis of PD. Recent studies suggest that 27–60% of all cases might be caused by genetic factors [3–5].

The gene encoding α-synuclein, *SCNA*, was the first gene identified causing autosomal recessive inherited PD when mutated, followed by the discovery of PARK2 and PINK1 [6–8]. Interestingly, all of these proteins are involved in macroautophagy (referred to herein as autophagy) processes, leading to the conclusion that autophagy plays an important role in PD. Indeed, studies have shown that the autophagic flux is profoundly disrupted in PD patients, whereas it remains a matter of debate whether this is a cause or result of the disease [9]. A large meta-analysis of genome-wide association studies of PD analyzed the genome of over 13,000 PD patients and compared it to more than 95,000 controls. Many new genes causative for PD were identified from this study, including *LRRK2*, *GBA*, *TMEM175*, *GPNMB*, *MAPT*, *SCARB2*, and *SREBF1*, each of which is directly or indirectly involved in autophagy [10].

Autophagy is a conserved catabolic cellular process during which macromolecules, organelles, and cytosol fractions are degraded by the lysosomes, which contain acid hydrolases (such as proteases, lipases, or nucleases) that break down the internalized macromolecules. Essential components of these macromolecules can be used for energy yield and the building of new cellular material. Although initially described as a stress-induced mechanism, autophagy exerts basal activity and has a major role in the quality control of proteins to maintain cellular homeostasis. The role of autophagy for neuroprotection and neurodegeneration in general is well established, and its stringent regulation is critical for healthy neuronal homeostasis [11]. For instance, autophagy can ameliorate symptoms of PD by removing aggregated proteins, whereas excessive autophagy in contrast may contribute to DA cell death [12].

Interestingly, impaired autophagy is also linked to schizophrenia [13], a mental illness that typically commences in young adulthood with a lifetime prevalence of about 0.5% [14]. Symptoms, such as abnormal social behavior and the inability to distinguish between reality and the imaginary, are usually attributable to disturbed dopamine signaling. In contrast to PD, where the lack of dopamine is the perpetrator, high dopamine levels and dopamine hyper-responsiveness seem to be responsible for schizophrenic symptoms [15].

A potential role in both PD and schizophrenia is attributed to the enzyme nicotinamide-N-methyltransferase (NNMT), which is expressed in all body tissues including the nervous system and represents a key player in NAD^+^/NADH metabolism. The central coenzyme for fuel oxidation and interconversion of different classes of metabolites is NAD^+^, which is typically reduced to NADH during these processes. It can furthermore be used by the sirtuins, which link lysine deacetylation to the turnover of NAD^+^, and poly ADP-ribose polymerases (PARPs) for DNA repair. One of the products of these reactions is nicotinamide (NAM). NAM is one of three NAD^+^ precursor vitamins (vitamin B3) and can be salvaged and used in re-synthesis of NAD^+^, or converted irreversibly by NNMT to N-methylnicotinamide (MNA), using S-adenosyl methionine (SAM) as methyl group donor [16]. NNMT has been shown to play a crucial role in obesity, as limiting re-synthesis of NAD^+^ decreased fuel oxidation, leading to fat storage in mice [17]. In contrast, NNMT in liver improves lipid parameters via sirtuin 1 stabilization to protect from some effects of high fat diet-induced obesity [18], pointing towards significant tissue-specificity of NNMT. The enzyme has furthermore been shown to play a crucial role in reactive oxygen species signaling and aging [19], and is highly expressed in cancer cells [20, 21], where it influences epigenetic regulation [22]. Several studies have implicated NNMT in PD, schizophrenia, and other neurological disorders such as bipolar disorder and epilepsy [23–28]. Notably, both PD and schizophrenia are associated with methylation disturbances in the cell [29, 30]. Although some *in vitro* studies have found potential mechanisms that contribute to NNMT action in neurons [31, 32], no mechanism of action has been described *in vivo* so far.

Here we investigate for the first time the neuronal role of NNMT in the context of neuronal homeostasis, behavior, neurodegeneration, and lifespan *in vivo* using the model organism *Caenorhabditis elegans*. This small nematode has provided valuable insights into the cellular mechanisms of neurodegeneration, neurological control of behavior, and aging. The *C*. *elegans* nervous system is simple, yet many of its structural features and the associated cellular and biochemical processes are very similar to those of most vertebrate nervous systems. *C*. *elegans* is the only organism whose neuronal wiring has been completely documented, showing surprisingly complex neuronal circuits and behavioral plasticity. Additionally, its short lifespan of about 4 weeks allows for the study of living, aging animals, which is an important consideration since age is a major risk factor for neurodegeneration.

*anmt-1*, the *C*. *elegans* ortholog of human *nnmt*, is naturally not expressed in the nervous system of the worm. We discovered that a mild ectopic neuronal expression of *anmt-1* regulates neurotransmitter production and neuronal autophagy via influencing the availability of intracellular methyl groups. Thus, ANMT-1 influences neuronal homeostasis, behavior, degeneration, and organismal health- and lifespan. ANMT-1 competes for methyl groups from SAM with another methyltransferase, LCMT-1, the worm ortholog of human LCMT1 (leucine carboxyl methyltransferase 1), as the methylation to MNA is irreversible, creating methylation drainage in the cell. Consequently, LCMT-1 activity is limited, leading to a switch in a pathway containing LET-92/PP2A (protein phosphatase 2) and NPRL-2/NPRL2 (human NPR2-like, GATOR1 complex subunit), which induces autophagy. We further show that the regulation of autophagy via ANMT-1 is beneficial in neurodegenerative disease models. Notably, in the case of neuronal autophagy dysfunction, high ANMT levels become a trigger for neurodegeneration. In summary, ANMT-1/NNMT is a pivotal element in neuronal cell metabolism that regulates neuronal homeostasis and may contribute to the prevalence of neurological disorders.

## Results

### *anmt-1* expression in the dopamine system influences neurodegeneration, lifespan, and fertility

NNMT in humans is suspected of being involved in the progression of PD, which is characterized by loss of DA neurons in the substantia nigra in the brain. Also, NNMT may play a role in mental disorders such as schizophrenia and bipolar disorder that both are characterized by disturbances in dopamine levels and signaling. In this context, we investigated the influence of neuronal ANMT-1 in the DA system of *C*. *elegans* by expressing *anmt-1* using the DA neuronal-specific *dat-1* promoter (*anmt-1*^dopa^) and the MosSCI cloning system, which results in a mild ectopic expression in the DA neuronal tissue. *anmt-1* is, in contrast to *nnmt* in humans, not expressed in the *C*. *elegans* nervous system [19]. To control for specific ANMT-1 activity we expressed a mutated version of *anmt-1* (*anmt-1*^dopa-MUT^) in the DA neurons, which contains point mutations in 6 conserved SAM binding sites (details can be found in *Methods*). A mild ectopic expression of *anmt-1* in the GABAergic motor neuronal system (*anmt-1*^GABA^) was used as control for DA-specific effects. **S1A Fig** depicts a transgenic animal that expresses GFP under the control of *dat-1* (GFP^dopa^), which was used to visualize the DA nervous system.

**Fig 1:**
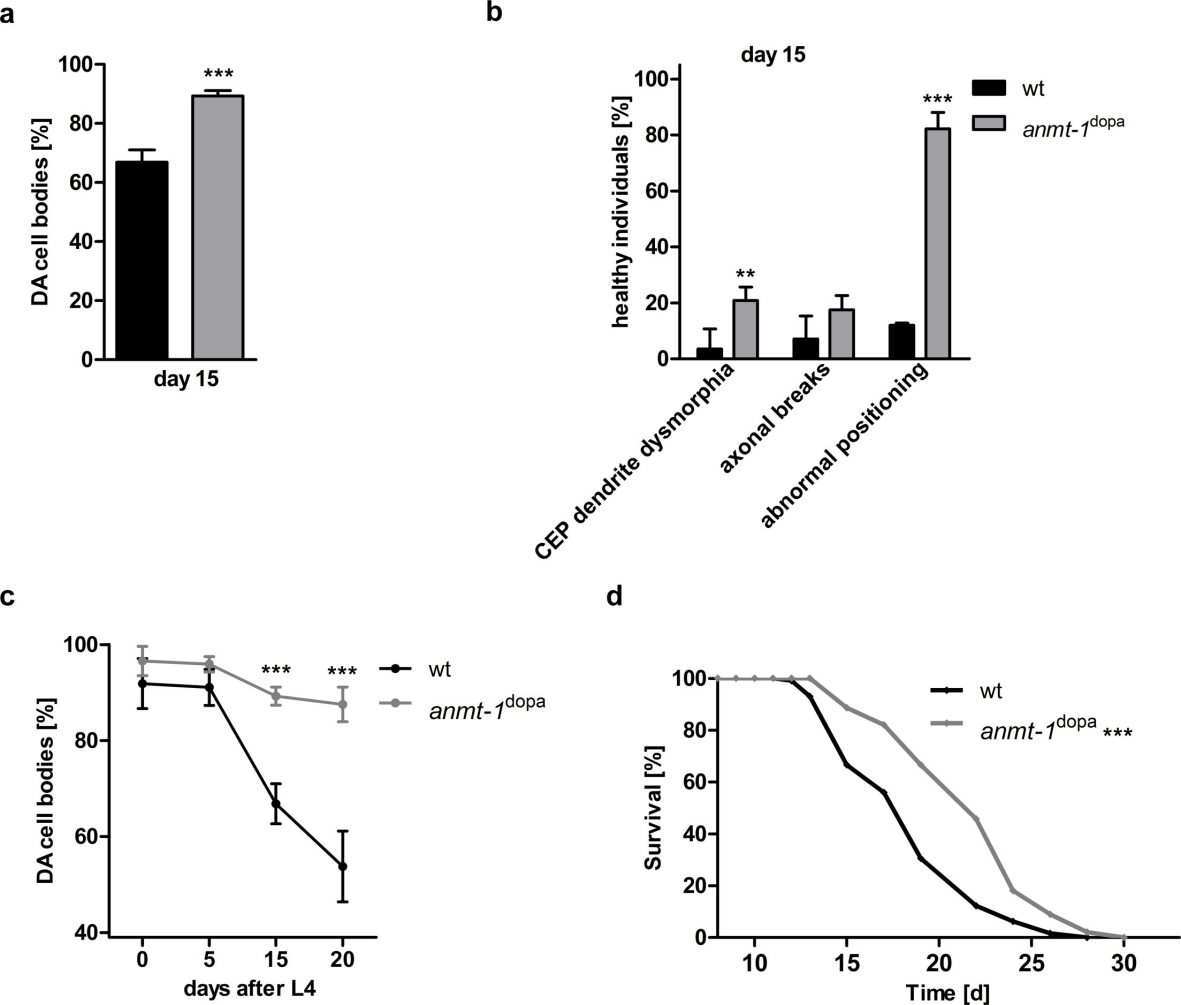
*anmt-1* expression in the dopamine system influences neurodegeneration and lifespan. **a** Presence of dopaminergic (DA) cell bodies in wt (black) and *anmt-1*^dopa^ (grey) at day 15 of adulthood. **b** DA neuronal morphology categorized in CEP dendrite dysmorphia, axonal breaks, and abnormal cell body and axon positioning in percentage of healthy individuals of wt and *anmt-1*^dopa^ at day 15 of adulthood. Note that the percentage of healthy worms regarding the different anomalies was depicted for easier comparison, so that in both the cell body and morphology graphs a completely healthy population equals 100%, and a completely sick population equals 0%. **c** DA cell body presence in wt and *anmt-1*^dopa^ at different ages. **d** Lifespan analysis of *anmt-1*^dopa^ compared to wt. **: p < 0.01, ***: p < 0.001.

As PD in humans is an age-dependent disease, we analyzed worms at day 15 after L4, which represents an old stage of life for these animals, and counted DA neuronal cell bodies. Interestingly, we found that the number of DA neurons in old *anmt-1*^dopa^ is significantly higher than in wt worms of the same age **(Fig 1A,** see **S1B Fig** for DA neurons broken down into CEP, ADE, and PDE cell subclasses). We also analyzed DA neuronal morphology by checking worms for CEP dendrite dysmorphia, axonal breaks, and abnormal cell body and axon positioning as shown in different stages of neurodegeneration in **S1A Fig**. In agreement with the DA cell body count, 15 days old *anmt-1*^dopa^ animals display a higher percentage of healthy individuals that do not show the above-mentioned morphological defects when compared to wt **(Fig 1B)**. Analyses of neurodegeneration phenotypes at different ages revealed that a tendency towards less morphological defects could be observed as early as day 5 of adulthood **(S1D Fig)**, whereas no differences were found for DA cell body quantity at young ages **(S1C and S1E Fig)**, **Fig 1C** depicts the presence of DA neurons in *anmt-1*^dopa^ and wt animals over time. Notably, the two investigated strains that express a mutated version of *anmt-1* (*anmt-1*^dopa-MUT 1 and 2^) did not show decreased neurodegeneration when compared to wt at day 15 of adulthood **(S1F Fig)**. Table 1 contains data from a pulse and chase experiment where *anmt-1*^dopa^ animals with an exclusively neuronal RNAi-sensitive background were treated with RNAi against *anmt-1*. Worms were put on *anmt-1* RNAi at different time points and microscopy for neurodegeneration was performed at day 15 of adulthood. The beneficial effect of neuronal *anmt-1* expression is completely abolished when worms were fed *anmt-1* RNAi beginning from the young adult stage as DA neuronal cell body quantity is indistinguishable from wt animals of the same age **(S1G Fig**; see Table 1 for other neurodegeneration phenotypes). Interestingly, *anmt-1*^dopa^ animals that were treated with *anmt-1* RNAi from hatching experience worse neurodegeneration than wt at day 15 **(S1G Fig)**, pointing towards a critical role for *anmt-1* during neurodevelopment.

**Table 1:** Effects of *anmt-1* RNAi on neurodegeneration at day 15 of adulthood in *anmt-1*^dopa^ with a neuronal RNAi-sensitive background.

Treatment	DA cells [%] +/-SD				Morphology [% of healthy individuals] +/-SD		
	all	CED	ADE	PDE	CEP dendrites	Axonal breaks	Abnormal positioning
*anmt-1*^dopa^, shifted to *anmt-1* RNAi at:							
Egg	46.6 +/- 3.4	61.9 +/- 3.3	25.9 +/- 5	36.6 +/- 7.6	0 +/- 0	0 +/- 0	26.2 +/- 2.1
L4/young adults	76.1 +/- 9.8	82.4 +/-15.7	51.2 +/- 9	88.4 +/- 1.5	0 +/- 0	4.2 +/- 4.8	29.4 +/- 6.9
Day 3	69.4 +/- 9.8	78.6 +/- 7.4	45.8 +/- 9	88.4 +/- 3.8	15.5 +/- 11.7	16.9 +/- 2.4	33.3 +/- 7.2
Day 5	74 +/- 9	81 +/-10.3	58.1 +/- 9.6	75.3 +/- 3.2	16.7 +/- 11.7	20.8 +/- 8.3	37.9 +/- 4.8
Day 8	78.2 +/- 1.6	77.6 +/- 5	61.9 +/-11.3	95.8 +/- 7.2	19.6 +/- 9.2	32.1 +/- 2.4	88.9 +/- 11.1
Day 10	86 +/- 1.6	84.7 +/- 4.4	81.1 +/- 2.4	93.5 +/- 7.2	29.9 +/- 9.2	24.3 +/- 5	87.5 +/- 12.5
Day 12	90.1 +/- 0.7	91.3 +/- 4.9	85.8 +/- 6.2	91.8 +/- 2.6	33.8 +/- 4.7	37.4 +/- 4.9	75 +/- 12.5
EV control	93.2 +/- 4.4	99.2 +/- 1.3	78.1 +/-15.3	96.3 +/- 6.4	51.4 +/- 2.8	45.8 +/- 10.2	87 +/- 3.2

The GABAergic nervous system, as visualized with an mCherry reporter (mCherry^GABA^; **S2A Fig)**, was not affected by *anmt-1*: No differences in GABAergic cell body and commissure number **(S2B and S2C Fig)**, and GABAergic axonal breaks **(S2D Fig)** were observed in *anmt-1*^GABA^ and wt at day 15. We wondered whether the lack of age-dependent neurodegeneration in *anmt-1*^dopa^ might also affect their longevity and found that indeed their lifespan is significantly extended compared to wt **(Fig 1D)**, whereas lifespan analyses in *anmt-1*^dopa-MUT 1 and 2^, transgenics that have mutations potentially disturbing ANMT-1 enzymatic function, did not yield such a result **(S1H Fig)**. *anmt-1*^dopa^ with a neuronal-sensitive background for RNAi treated with *anmt-1* RNAi lived significantly shorter than *anmt-1*^dopa^ fed with control RNAi **(S1I Fig)**, suggesting that indeed the ectopic *anmt-1* expression in DA neurons is responsible for the observed longevity. Surprisingly, *anmt-1*^GABA^ transgenics also showed a slight lifespan extension **(S2E Fig)**, although no effect on GABAergic neurodegeneration was observed. Furthermore, fertility tests revealed a reduced brood size in *anmt-1*^dopa^ **(S1J Fig)**, but not in *anmt-1*^GABA^ animals **(S2F Fig)**. These data suggest that DA neuronal ANMT-1 signaling regulates not only neurodegeneration and aging, but also influences reproduction, suggesting that ANMT-1 may act in an endocrine or neuroendocrine manner.

Previous research by us and other laboratories has shown that some of the beneficial effects of ANMT-1/NNMT are due to elevated concentrations of MNA, the product of NAM methylation [16]. We wondered whether this is also the case in neurodegeneration and incubated wt worms with 1 μM MNA, a concentration that has been shown previously to be lifespan-extending in *C*. *elegans* [19]. At 15 days after L4, these worms showed no significant differences in DA neuron number **(S1K Fig)** and morphology of the DA system compared to controls **(S1L Fig)**. Thus, we concluded that increasing MNA levels alone are insufficient for the beneficial effects of neuronal *anmt-1* expression.

### *anmt-1*^dopa^ expression affects behavior and dopamine levels, but influences neurodegeneration and lifespan independently of classic dopamine signaling

**Fig 2:**
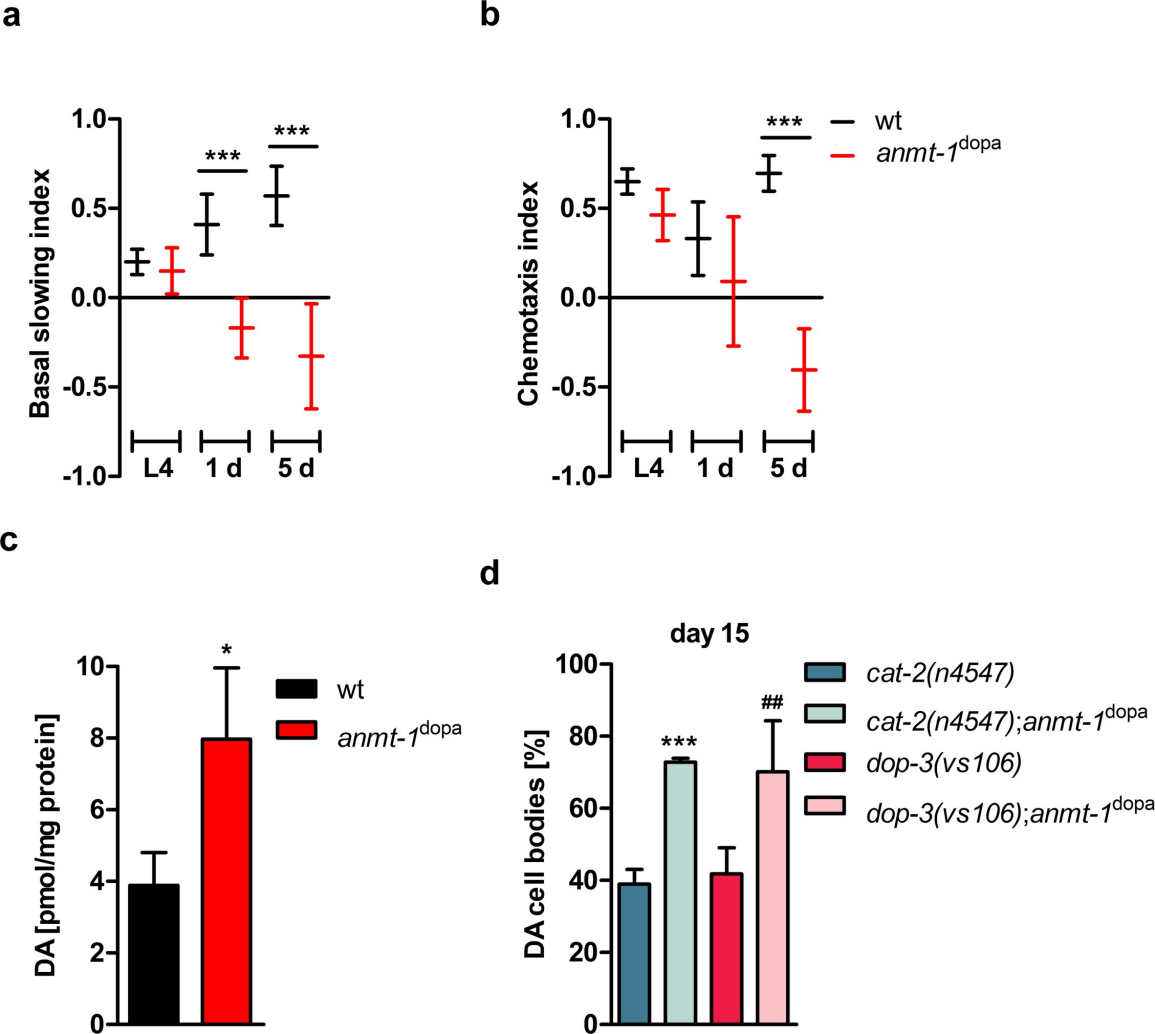
***anmt-1***^**dopa**^ **expression affects behavior and dopamine levels, but influences neurodegeneration and lifespan independently of dopamine signaling.a and b** Dopamine-dependent behaviors in wt (black) and *anmt-1*^dopa^ (red) at L4, L4 + 1 day, and L4 + 5 days of adulthood. **a** Basal slowing index. **b** Chemotaxis index based on ethanol avoidance behavior. **c** Dopamine concentration in pmol/mg protein of a mixed population of wt and *anmt-1*^dopa^. **d** Presence of (DA) cell bodies in *cat-2(n4547);anmt-1*^dopa^ (light turquoise) compared to *cat-2(n4547)* (turquoise), and *dop-3(vs106)*;*anmt-1*^dopa^ (light pink) compared to *dop-3(vs106)* (pink) at day 15 of adulthood. *: p < 0.05, ^##^: p < 0.05, ***: p < 0.001, d * compared to *cat-2(n4547)*, ^#^ compared to *dop-3(vs106)*.

Since we found DA neurodegeneration modulated by ANMT-1, we wondered whether DA neuronal *anmt-1* expression influences dopamine-dependent behaviors in *C*. *elegans*, possibly resembling features of schizophrenia in humans, which is characterized by dysfunctional dopamine signaling. Therefore, we tested two common dopamine-dependent behaviors in *anmt-1*^dopa^ animals, namely the abilities to sense food and ethanol [33, 34]. Briefly, when *C*. *elegans* is kept without food, they survey their environment for a potential food source. The mechanic stimulus of bacteria will make them slow down and they will remain on the food rather than moving on to other areas. This so-called “basal slowing response” is mediated by DA neurons and dopamine signaling, as is the avoidance response to ethanol. At 1 and 5 days after L4, *anmt-1*^dopa^ worms do not stick to a discovered food source like wt worms **(Fig 2A)**. Furthermore, they do not actively avoid the smell of ethanol at day 5 **(Fig 2B)**. It is interesting to note that these behavioral abnormalities manifest only in adult worms, as they are not present in L4 larvae **(Fig 2A and 2B)**. The fact that these behavioral assays rely on movement prevented us from examining older animals that are less mobile. We therefore tested basal slowing employing a different approach according to [35]. In short, we counted body bends of worms washed free of bacteria and put either on empty NGM plates or plates seeded with OP50. Wt animals had a significantly lower number of body bends when put on OP50 than on empty plates at day 1 **(S3A Fig)**, 5 **(S3B Fig)**, and 10 **(S3C Fig)** of adulthood. This was not the case in *anmt*^dopa^ transgenics as their movement was the same on empty plates and plates with bacteria, confirming the phenotype of Fig 2A. The behavior of *anmt-1*^dopa-MUT 1 and 2^, however, resembled as expected that of wt worms, because ANMT-1 is not functional **(S3D Fig)**. Additional phenotypes related to impaired dopamine signaling, such as swimming-induced paralysis, could not be observed. *C*. *elegans* locomotion is GABA-dependent, thus we examined locomotion in *anmt-1*^GABA^ transgenics, but we did not observe any differences compared to wt controls in either 5 or 10 days old worms **(S2G and S2H Fig)**.

Abnormal dopamine-dependent behaviors have been observed previously in *C*. *elegans* with low dopamine levels, and could be rescued with external dopamine [36]. Therefore, we incubated worms with 50 mM dopamine for 4 to 6 hours before basal slowing response and ethanol avoidance assays were performed, which however did not change either of these behaviors **(S3E and S3F Fig)**.

We then tested dopamine and GABA concentration in *anmt-1*^dopa^ and *anmt-1*^GABA^, respectively. A significant increase to 8 ± 2 pmol dopamine per mg protein was found in *anmt-1*^dopa^ compared to wt animals with 3.9 ± 0.9 pmol/mg **(Fig 2C)**. These elevated levels explain why additional external dopamine could not correct the dopamine-dependent behaviors. *anmt-1* overexpressed in GABAergic neurons had no impact on GABA concentration **(S2I Fig)**.

Because of the increase dopamine levels in *anmt-1*^dopa^, we wondered whether the longevity and decreased neurodegeneration of this strain was dependent on dopamine synthesis and signaling. Therefore, we crossed *anmt-1*^dopa^ animals into *cat-2(n4547)* worms, which have a loss-of-function deletion in the gene that encodes the tyrosine hydroxylase CAT-2, the key enzyme in dopamine synthesis [36]. *cat-2(n4547)*;*anmt-1*^dopa^ animals do not show increased lifespan compared to *cat-2(n4547)* controls **(S3G Fig)**. *C*. *elegans* has at least three dopamine receptors. DOP-1 and DOP-3, homologs of mammalian D1 and D2 dopamine receptors, that work together antagonistically to regulate dopamine-dependent locomotion, whereas a function for DOP-2 has not yet been determined [35]. In contrast to disabled dopamine synthesis, *anmt-1*^dopa^ animals with a deletion in *dop-3* (*dop-3(vs106)*;*anmt-1*^dopa^), hence disturbed dopamine signaling, still show lifespan extension **(S3H Fig)**, suggesting that dopamine production and elevated levels may mediate longevity, rather than DOP-3 signaling.

We also tested neurodegeneration in old (15 d) *cat-2(n4547)* and *dop-3(vs106)* animals and found that *anmt-1*^dopa^ is still able to prevent loss of DA neurons in *cat-2(n4547)*;*anmt-1*^dopa^ and *dop-3(vs106)*;*anmt-1*^dopa^ **(Figs 2D and S3I)**, therefore acting independently of CAT-2 and DOP-3 in regards to neuroprotection.

### Neuronal autophagy mediates *anmt-1*^dopa^ induced phenotypes

Since neither the ANMT-1/NNMT metabolite MNA, nor dopamine signaling was responsible for the neuroprotective effect of DA neuronal *anmt-1* expression, we wondered if the methylation itself that is catalyzed by ANMT-1/NNMT influences neuronal cell metabolism. ANMT-1/NNMT uses SAM as methyl group donor, and as the reaction to MNA is irreversible, the methyl groups used cannot be recycled to recreate SAM, a mechanism that is highly conserved through evolution [22]. It has been reported in yeast that decreasing SAM levels act as stress and/or starvation signal for the cell to induce autophagy [37, 38]. Autophagy is a tightly regulated catabolic process within cell metabolism to degrade misfolded proteins and damaged macromolecules, and dysregulation resulting in too high or too low levels is observed in PD and schizophrenia [9, 13]. We hypothesized that neuronal ANMT-1/NNMT activates autophagy via decreasing SAM levels. Overly active autophagy could be problematic in early life and the abnormal behavior we observed in young adult animals might be a result of excessive autophagy. In contrast, these same autophagy levels might be of advantage later, degrading misfolded proteins and dysfunctional macromolecules as they increase with age. Since the behavioral phenotypes are not accompanied by DA neurodegeneration at older stages, we hypothesized that elevated autophagy helps to maintain neuronal health and extends lifespan in *anmt-1*^dopa^ animals.

**Fig 3:**
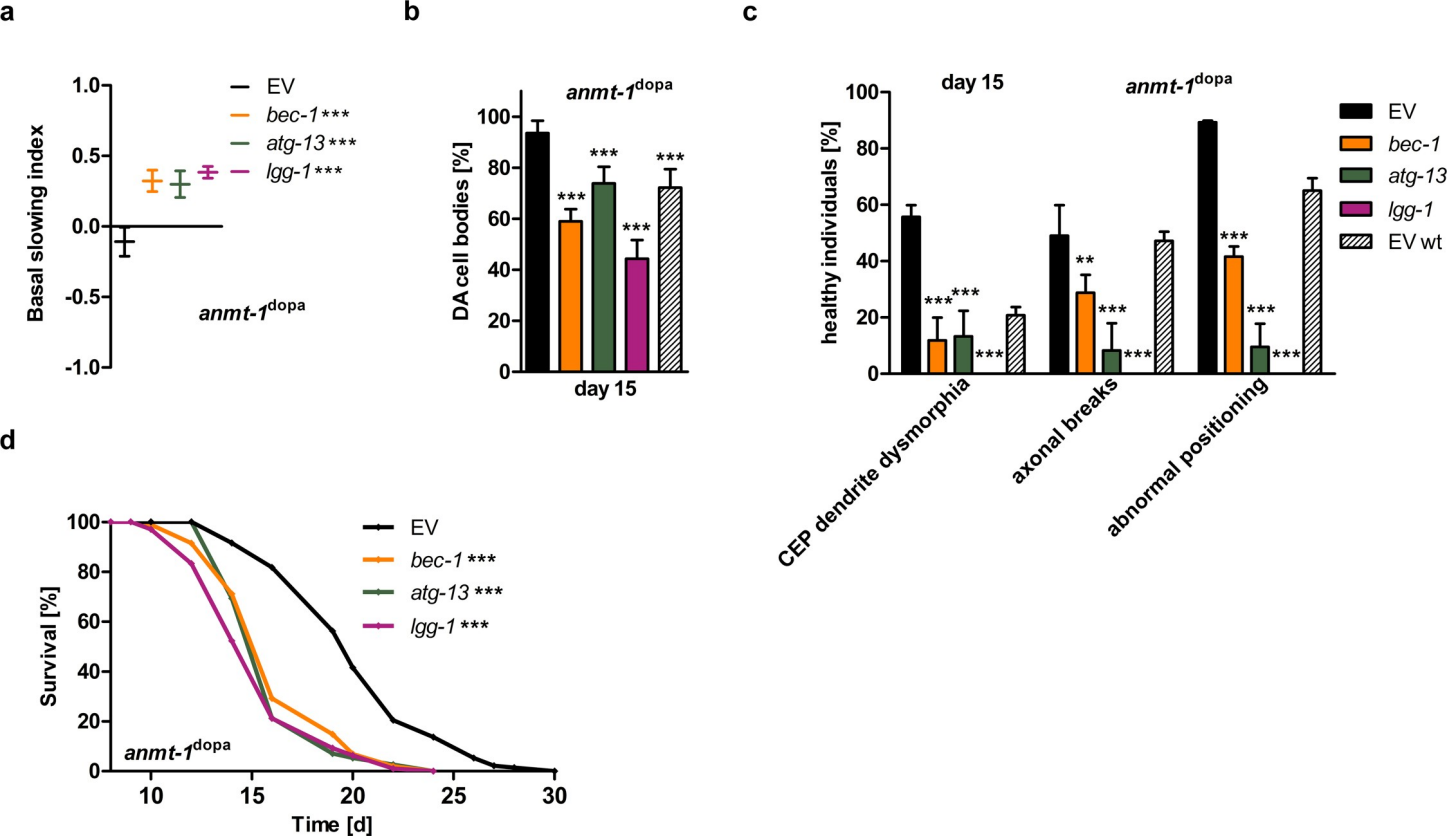
Neuronal autophagy mediates *anmt-1*^dopa^-induced phenotypes. **a** Basal slowing index of *anmt-1*^dopa^ with a neuronal RNAi-sensitive background treated with RNAi against *bec-1* (orange), *atg-13* (green), or *lgg-1* (purple) compared to control RNAi (EV; black) at day 5 of adulthood. **b** Presence of (DA) cell bodies in *anmt-1*^dopa^ with a neuronal RNAi-sensitive background treated with RNAi against *bec-1*, *atg-13*, or *lgg-1* compared to EV and wt EV (patterned) at day 15 of adulthood. **c** DA neuronal morphology in *anmt-1*^dopa^ with a neuronal RNAi-sensitive background treated with RNAi against *bec-1*, *atg-13*, or *lgg-1* compared to EV and wt EV at day 15 of adulthood. **d** Lifespan of *anmt-1*^dopa^ with a neuronal RNAi-sensitive background treated with RNAi against *bec-1*, *atg-13* and *lgg-1* compared to EV. **: p < 0.01, ***: p < 0.001.

We tested whether the phenotypes we observed in *anmt-1*^dopa^ worms could be reversed by inhibiting neuronal autophagy. We achieved this by knocking down the neuronal expression of essential genes driving autophagy (*bec-1*/*BECN1*, *atg-13*/*ATG13*, *lgg-*1/*MAP1LC3A*) using RNAi in an exclusively neuronal RNAi-sensitive background. *bec-1* and *atg-13* are critical for initiating autophagy, and *lgg-1* is important at the later stage of autophagosome formation [39]. Applying RNAi against each of these genes rescued the abnormal basal slowing response of *anmt-1*^dopa^ animals at 5 days of adulthood **(Fig 3A)** and abolished the neuroprotective effect on DA cell body maintenance and morphology at day 15 **(Figs 3B, 3C and S4A)**. Similar treatment had no effect, or was even beneficial in regards to neurodegeneration, in control animals **(S4B, S4C and S4D Fig)**. Notably, the neuroprotective effect of *anmt-1*^dopa^ is not only abolished when these worms experience a knockdown in neuronal autophagy genes, but *anmt-1*^dopa^ show an increased loss of DA cells and a higher degree of morphological defects compared to wt under these circumstances **(Fig 3B and 3C)**. The same effect in *anmt-1*^dopa^ was observed at day 5 **(S4E and S4F Fig)**, whereas the contrary could be found in wt **(S4G and S4H Fig)**. This suggests a secondary increase in neurodegeneration due to DA neuronal *anmt-1* expression when autophagy is dysfunctional, which could contribute to PD progression under these circumstances.

We wondered if these results are reflected in life expectancy and thus, analyzed lifespan of both neuronal RNAi-sensitive *anmt-1*^dopa^ and wt animals treated with *bec-1*, *atg-13* and *lgg-1* RNAis. As expected, the above-mentioned RNAis decreased lifespan significantly in *anmt-1*^dopa^ animals **(Fig 3D)**, strikingly well below wt life expectancy. In wt, neuronal loss of *bec-1* and *atg-13* slightly extended lifespan, while *lgg-1* RNAi decreased lifespan **(S4I Fig)**.

In sum, these data suggest that the behavioral changes, neuroprotection, and lifespan extension observed in *anmt-1*^dopa^ animals is dependent on neuronal autophagy. This beneficial effect on neuronal health and lifespan is reversed when autophagy is depleted, revealing a potential mechanism of how high *anmt-1*/*NNMT* expression could increase PD risk.

### ANMT-1 regulates autophagy by controlling SAM levels

To investigate more directly whether ANMT-1/NNMT is able to regulate autophagy, we used a reporter strain in which LGG-1 is tagged with mCherry [P_*nhx-2*_::*mCherry*::*lgg-1*]. LGG-1 occurs diffusely in the cytosol under non-stressed conditions. Autophagy induction leads to LGG-1 organisation around autophagosomes, which then become visible and quantifiable as puncta. To induce autophagy, we starved worms for about 24h prior quantification and observed an increase in the average number of autophagosomes/puncta per wt worm, ranging from 34.4 ± 12.9 to 110 ± 35.5 in 1 day old adults **(S5A and S5B Fig)**. We also used pimozide as positive control, since it has been identified as autophagy inducer in *C*. *elegans* [40], and the potent autophagy inhibitor 3-methyladenine in starved worms as negative control [41] **(S5A Fig)**. To investigate whether autophagy levels change during aging, we determined basal autophagy levels in wt animals in 5 and 10 days old adults. Puncta quantity was similar in 5 days old worms versus 1 day old, but later increased significantly to 107.7 ± 25.3 per animal at day 10 of adulthood **(S5C Fig)**. Strong background fluorescent prevented us from quantifying puncta at later time points. Interestingly, the increase in puncta formation due to starvation appeared to be progressively dysregulated with aging, as the increase in young adults was > 3-fold when compared to fed animals, > 2-fold at day 5, and no increase at all could be detected at day 10 **(S5C Fig)**.

**Fig 4:**
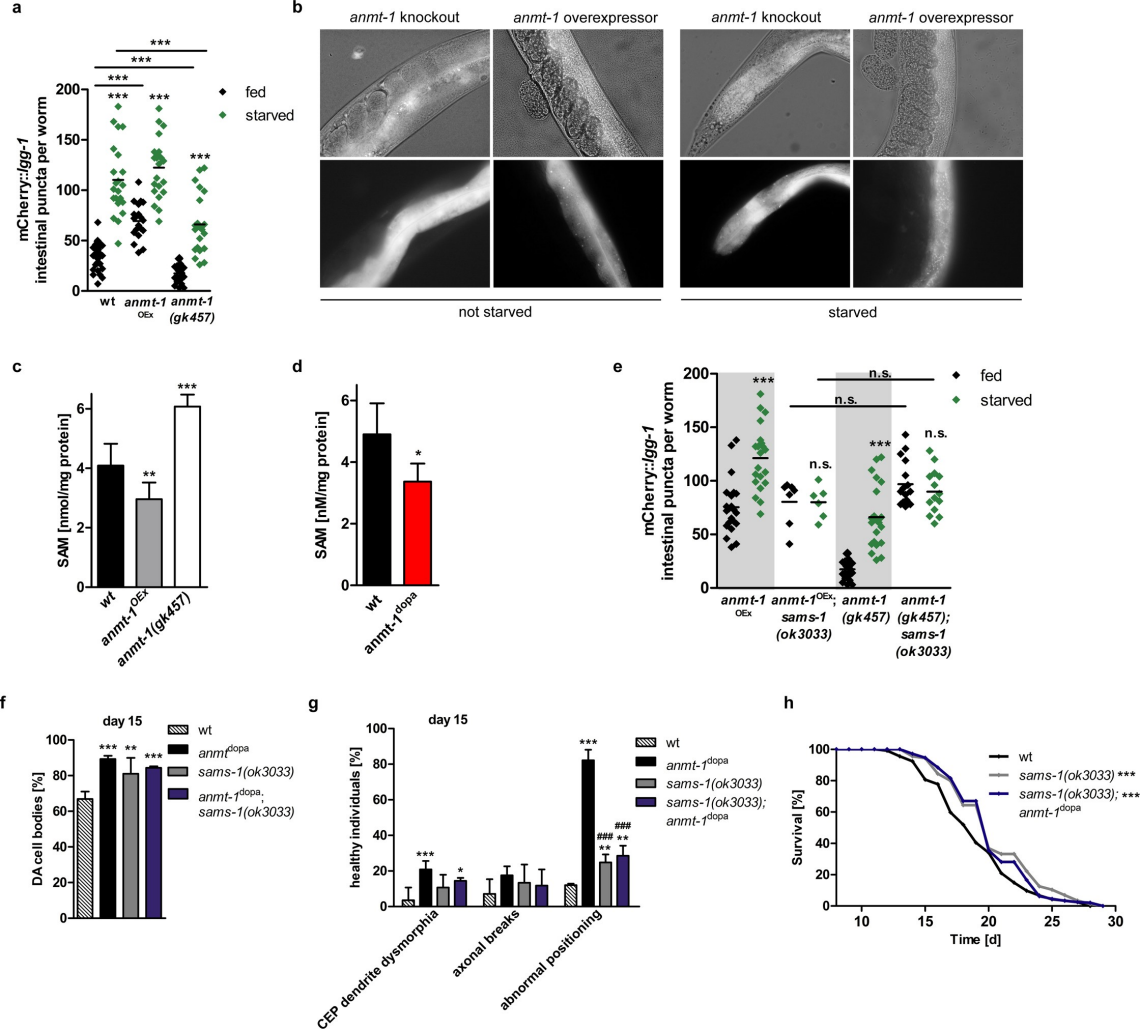
ANMT-1 regulates autophagy by controlling SAM levels. **a** Number of puncta per individual worm in wt, *anmt-1*^OEx^ and *anmt-1(gk457)* with a P_*nhx-2*_::mCherry::*lgg-1* background as marker for autophagosome formation after feeding (black) or 24 h of starvation (green). **b** Representative pictures of fed and starved wt, *anmt-1*^OEx^ and *anmt-1(gk457)* with a P_*nhx-2*_::mCherry::*lgg-1* background under white light and fluorescence light. **c** S-adenosylmethionine (SAM) concentration in nmol/mg protein of a mixed population of wt (black), *anmt-1*^OEx^ (grey), and *anmt-1(gk457)* (white). **d** SAM concentration in nmol/mg protein of a mixed population of wt and *anmt-1*^dopa^ (red). **e** Number of puncta per individual worm in *anmt-1*^OEx^ and *anmt-1(gk457)* as controls (underlaid in grey), *anmt-1*^OEx^;*sams-1(ok3033)* and *anmt-1(gk457)*;*sams-1(ok3033)* at day 1 of adulthood after feeding or 24 h of starvation. **f** Presence of (DA) cell bodies in *sams-1(ok3033)* (grey) and *anmt-1*^dopa^*;sams-1(ok3033)* (dark blue) compared to wt (patterned) and *anmt-1*^dopa^ (black) at day 15 of adulthood. **g** DA neuronal morphology in *sams-1(ok3033)* and *anmt-1*^dopa^*;sams-1(ok3033)* compared to wt and *anmt-1*^dopa^ at day 15 of adulthood. **h** Lifespan of *sams-1(ok3033)* and *anmt-1*^dopa^*;sams-1(ok3033)* compared to wt. *: p < 0.05, **: p < 0.01, ***^/###^: p < 0.001, n.s.: not significant; **g** ^#^ compared to *anmt-1*^dopa^.

Although LGG-1 puncta do occur in neurons in general, quantification in the DA neurons is difficult, as number and size of these cells are too small to gain evaluable results. We therefore decided to address this aspect in the whole animal instead of only DA neurons. A strain that overexpresses *anmt-1* under the control of its endogenous promoter (P_*anmt-1*_::*anmt-1*; *anmt-1*^OEx^), as well as *anmt-1*(*gk457*), a loss-of-function deletion mutant of *anmt-1*, were crossed into the mCherry::*lgg-1* autophagy reporter strain and puncta in whole worms were quantified at various time points. We found that basal levels of autophagy were significantly higher in *anmt-1*^OEx^ transgenics and significantly lower in *anmt-1*(*gk457*) mutants **(Fig 4A and 4B)**. When worms were starved for 24h to induce autophagy the same significant differences could be observed **(Fig 4A and 4B)**, suggesting that ANMT-1 regulates autophagy proportionally to its expression.

Subsequently, we investigated whether SAM is the mediator between ANMT-1 and autophagy. Since ANMT-1 uses methyl groups from SAM, we hypothesized it regulates its cellular levels. We analyzed SAM concentrations depending on *anmt-1* expression and found significantly lower SAM levels in *anmt-1*^OEx^ worms, and significantly higher levels in *anmt-1(gk457)* mutants **(Fig 4C)**, suggesting that ANMT-1 indeed regulates cellular SAM concentration. SAM levels were also analyzed in *anmt-1*^dopa^ whole animals and lower concentrations were found compared to wt **(Fig 4D)**, which is surprising here given that *anmt-1* is overexpressed in only eight cells of the animal. When SAM is used in a metabolic reaction, it is hydrolyzed to yield homocysteine. Homocysteine is converted either to cysteine or via tetrahydrofolate into methionine, which leads to recycling of SAM [42]. When methyl groups are not limited in the cell, SAM and homocysteine reciprocally regulate each other; *i*.*e*. high SAM causes low homocysteine, and vice versa. In *anmt-1(gk457)* worms SAM is not used by ANMT-1 and its levels increase **(Fig 4C)**, whereas homocysteine expectedly decreases **(S5D Fig)**. In *anmt-1*^OEx^ and *anmt-1*^dopa^, however, both SAM and homocysteine levels are decreased **(Figs 4C and 4D and S5D and S5E)**, suggesting the cycle is out of balance and methyl groups do indeed appear lost from metabolism.

In *C*. *elegans*, the majority of SAM is synthesized from methionine by S-adenosyl methionine synthetase (SAMS-1), which is encoded by the *sams-1* gene [43]. *sams-1(ok3033)* animals carry a major deletion in *sams-1*, leading to loss of function of the protein. We found that SAM concentration in *sams-1(ok3033)* mutants is with 2.3 nM/mg protein about 50% lower than in wt worms **(S5F Fig)**. As expected given the low SAM levels, we found significantly increased puncta numbers in *sams-1(ok3033)* compared to wt, suggesting that *sams-1(ok3033)* mutants have increased levels of autophagy **(S5G Fig)**. Notably, starvation did not further elevate puncta formation, consistent with a dysregulation of autophagic processes in the absence of *sams-1* and reduced SAM.

To directly address whether varying SAM levels are indeed responsible for autophagy regulation due to ANMT-1, we deprived *anmt-1*^OEx^ and *anmt-1(gk457)* animals genetically from SAM by crossing them with *sams-1(ok3033)* mutants. We found the regulation of autophagosome formation through *anmt-1* completely abolished, as indicated by *anmt-1*^OEx^ and *anmt-1(gk457)* mutants showing the same autophagy levels in a *sams-1(ok3033)* background **(Figs 4E, S5H and S5I)**. On a side note, we observed that *anmt-1(gk457)*;*sams-1(ok3033)* double mutants were completely sterile, suggesting an important developmental aspect of this pathway. Similarly, *anmt-1*^OEx^;*sams-1(ok3033)* animals did not have progeny that were homozygous for both the transgene and the mutation, indicating that the overexpression of *anmt-1* is lethal when SAM abundance is reduced or limited.

To examine whether the phenotypes observed in *anmt-1*^dopa^ are dependent on SAM, we analyzed the presence of DA neurons and morphology at day 15, as well as lifespan in *anmt-1*^dopa^;*sams-1(ok3033)*. *sams-1(ok3033)* seems to be neuroprotective for DA neurons and increases lifespan compared to wt **(Fig 4F, 4G and 4H)**. However, *anmt-1*^dopa^ could not further increase these beneficial effects **(Fig 4F, 4G and 4H)**, suggesting that the neuroprotection and longevity caused by *anmt-1*^dopa^ and loss of *sams-1* share a common mechanism, which include the reduction of SAM availability, hence activating autophagy.

### ANMT-1 regulates autophagy via the NPRL-2/NPRL2 pathway

**Fig 5:**
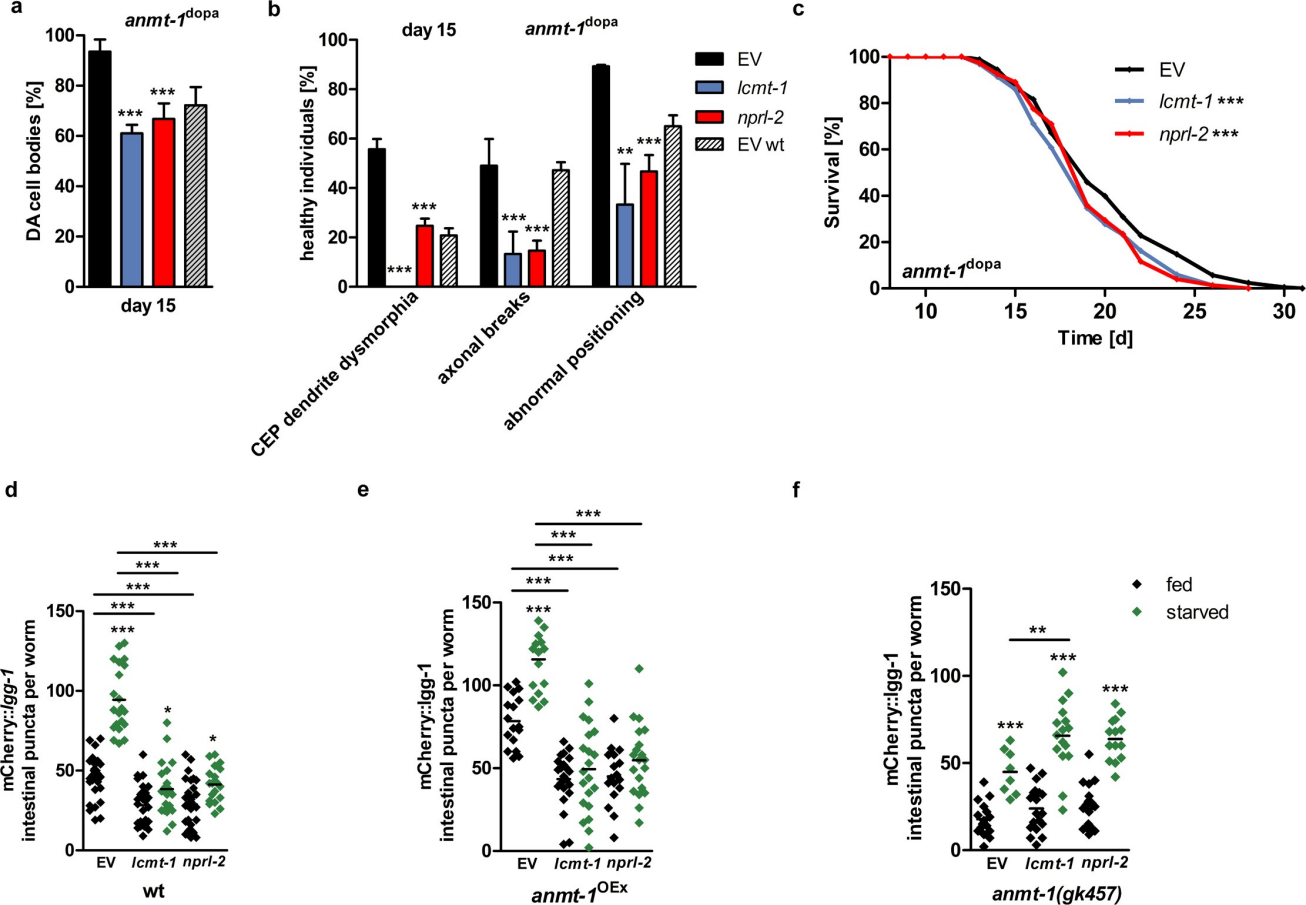
ANMT-1 regulates autophagy via the NPRL-2 pathway. **a** Presence of (DA) cell bodies in *anmt-1*^dopa^ with a neuronal RNAi-sensitive background treated with RNAi against *lcmt-1* (blue) and *nprl-2* (red) compared to control RNAi (EV; black) and wt EV (patterned) at day 15 of adulthood. **b** DA neuronal morphology in *anmt-1*^dopa^ with a neuronal RNAi-sensitive background treated with RNAi against *lcmt-1* and *nprl-2* compared to EV and wt EV at day 15 of adulthood. **c** Lifespan of *anmt-1*^dopa^ with a neuronal RNAi-sensitive background treated with RNAi against *lcmt-1* and *nprl-2* compared to EV. **d–f** Number of puncta per individual worm at day 1 treated with RNAi against *lcmt-1* and *nprl-2* compared to EV at day 1 of adulthood after feeding (black) or 24 h of starvation (green) in **d** wt, **e** *anmt-1*^OEx^ and **f** *anmt-1(gk457)*. **: p < 0.01, ***: p < 0.001.

In yeast, SAM regulates autophagy through the methyltransferase Ppm1p, an evolutionary conserved enzyme with orthologs in humans (leucine carboxyl methyltransferase, LCMT1) and *C*. *elegans* (LCMT-1). Ppm1p uses methyl groups provided by SAM to methylate and therefore activate the catalytic subunit of PP2Ap (protein phosphatase 2A in humans, LET-92 in *C*. *elegans*). Methylated PP2A then induces dephosphorylation of Npr2p (human NPR2-like, GATOR1 complex subunit and *C*. *elegans* NPRL-2), which is part of a complex that controls autophagy and cell growth via the regulation of TORC1, and potentially others [37, 44]. In *C*. *elegans*, the pathway is reportedly involved in reproduction and development [45] whereas its neuronal role has not been further investigated. However, it is known that *let-92* is highly expressed in the neurons [46]. We speculate that ANMT-1 competes with LCMT-1 for methyl groups from SAM, impairing the methylation ability of LCMT-1. This leads to decreased methylation and activity of LET-92, which can no longer dephosphorylate NPRL-2, thus inducing autophagy. Indeed, neuronal-specific RNAi downregulation of the genes involved in the pathway, *lcmt-1* and *nprl-2*, in *anmt-1*^dopa^ animals led to decreased DA cell body number at day 15 **(Figs 5A and S6A)**, and was milder at day 5 **(S6B Fig)**. In wt, the same treatment caused no, or a slightly beneficial, effect on DA neuronal loss at day 5 and 15 **(S6C and S6D Fig)**. Morphological damage was strongly increased by neuronal loss of *lcmt-1* and *nprl-2* in *anmt-1*^dopa^ at day 5 **(S6E Fig)** and day 15 **(Fig 5B)**, but decreased in wt at day 5 and 15 **(S6F and S6G Fig)**.

Both neuronal *lcmt-1* and *nprl-2* RNAi had a slight lifespan shortening effect in *anmt-1*^dopa^ animals **(Fig 5C)** in contrast to wt, where *lcmt-1* RNAi had no effect and *nprl-2* RNAi extended lifespan **(S6H Fig)**. We were unable to test the contribution of PP2A as it is an essential gene in *C*. *elegans*, and even RNAi applied only from L4 in an exclusively neuronal-specific RNAi-sensitive background had many non-specific effects that confounded phenotypic analyses.

The data obtained from neuronal *lcmt-1* and *nprl-2* loss resemble the results when autophagy was blocked by *bec-1*, *atg-13* and *lgg-1* RNAi **(Figs 3 and S4)**, suggesting that knocking down *lcmt-1* and *nprl-2* might indeed affect autophagy. Subsequently, the mCherry::*lgg-1* autophagy reporter strain was grown on *lcmt-1* and *nprl-2* RNAi and a downregulation of basal autophagy under feeding conditions compared to the control RNAi was found, as puncta quantity was significantly lower between the groups **(Fig 5D)**. In 1 day old adults, a small increase from 28.2 ± 13/28.9 ± 14 puncta per animal under feeding conditions to 37.7 ± 13.1/41.3 ± 10.8 puncta following starvation was observed when worms were grown on *lcmt-1* and *nprl-2* RNAi, respectively **(Fig 5D)**. At day 5 this effect was completely abolished **(S6I Fig)**, suggesting that *lcmt-1* and *nprl-2* play an important role in regulating starvation-induced autophagy. The same pattern was observed in *anmt-1*^OEx^, where RNAi against *lcmt-1* and *nprl-2* was able to completely abolish starvation-induced autophagy in 1 day old adults **(Fig 5E)**. Interestingly, knocking down *lcmt-1* and *nprl-2* also led to downregulation of basal autophagy levels of *anmt-1*^OEx^ back to wt levels **(S6J Fig)**. Strikingly, in *anmt-1(gk457)* mutants starvation could still induce autophagy when grown on *lcmt-1* and *nprl-2* RNAi **(Fig 5F)**, suggesting that the pathway including *lcmt-1* and *nprl-2* exclusively regulates autophagy in the presence of ANMT-1.

### *anmt-1* regulates *sams-1* and autophagy gene expression

**Fig 6:**
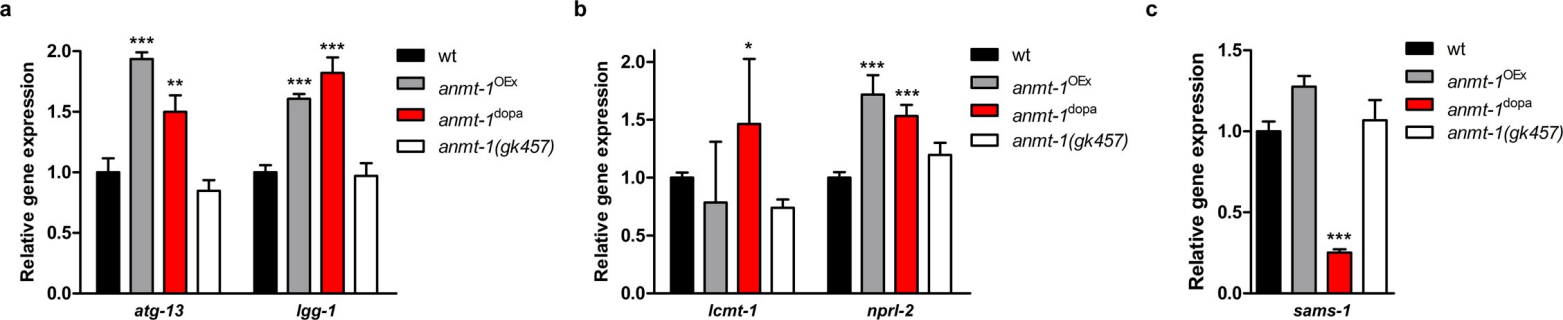
*anmt-1* regulates *sams-1* and autophagy gene expression. **a–c** Relative gene expression in a mixed population of wt (black), *anmt-1*^OEx^ (grey), *anmt-1*^dopa^ (red) and *anmt-1(gk457)* (white) of **a** *atg-13* and *lgg-1*, **b** *lcmt-1* and *nprl-2*, and **c** *sams-1*. *: p < 0.05, **: p < 0.01, ***: p < 0.001.

Autophagy is known to be subject to epigenetic regulation, and NNMT has been shown to regulate epigenetic processes by modifying SAM concentrations, influencing the progression of tumorigenesis [22, 47]. Whereas the epigenetic link between NNMT and autophagy is beyond the scope of this study and needs further investigation, we wondered how autophagy and *sams-1* gene expression is influenced by *anmt-1*. Notably, besides post-translational regulation, an important role for transcriptional control of autophagy has been described in recent research [47, 48]. Thus, we analyzed gene expression of *atg-13* and *lgg-1* in mixed populations of *anmt-1*^OEx^, *anmt-1*^dopa^, and *anmt-1(gk457)* animals. We found a stable upregulation of both genes by *anmt-1*^OEx^ and *anmt-1*^dopa^ compared to wt, whereas *anmt-1(gk457)* had no effect **(Fig 6A)**. Since ANMT-1 controls autophagy via the *lcmt-1/nprl-2* pathway, we sought to test the expression of these genes and found that *nprl-2* was upregulated by both *anmt-1*^OEx^ and *anmt-1*^dopa^, suggesting that ANMT-1-induced autophagy is regulated both post-translationally and transcriptionally **(Fig 6B)**. However, the results of *lcmt-1* gene expression showed pronounced differences between the experiments, leading to high standard deviations **(Fig 6B)**, which is consistent with a potential post-translational nature of LCMT-1 activity regulation via SAM. Surprisingly, we also found a strong transcriptional downregulation of *sams-1* only through *anmt-1*^dopa^ **(Fig 6C)**. This could mean that lower SAM levels in *anmt-1*^dopa^ are not exclusively due to metabolic consumption, but could also be regulated on a transcriptional level, potentially through epigenetic effects. Notably, all tested genes were regulated by *anmt-1*^dopa^. We hypothesize that the effect of gene regulation in only the eight DA neurons would be too small to detect in whole animals, thus again hinting towards an endocrine-like signaling of *anmt-1*^dopa^ that effects not only the nervous system, but potentially the whole organism.

In summary, these data reveal an alternative pathway of SAM and autophagy regulation in addition to post-translational/metabolic regulation, involving control on the transcriptional level, possibly through epigenetic processes.

### *anmt-1*^dopa^ rescues DA neurodegeneration in toxin-induced and genetic *C*. *elegans* models of PD

**Fig 7:**
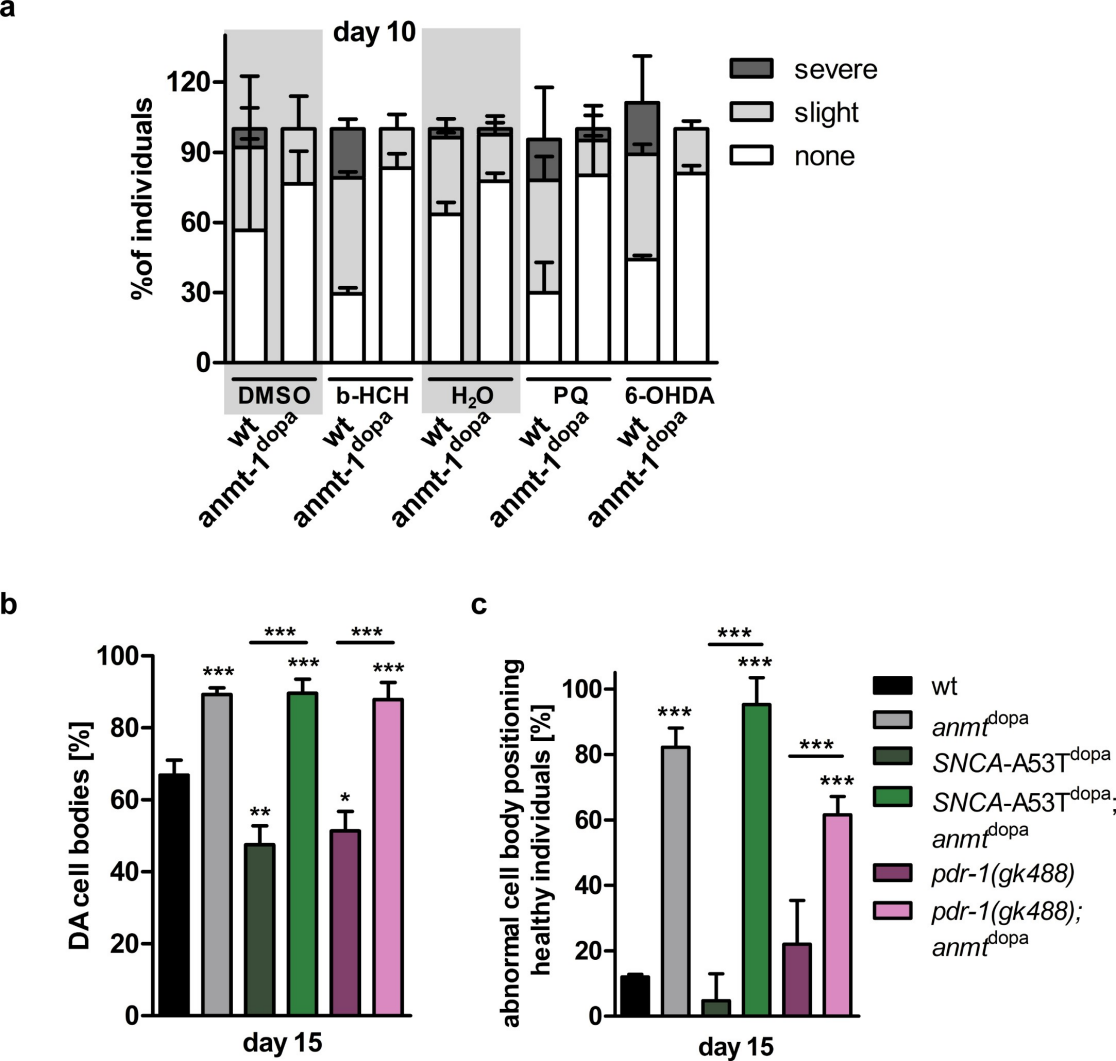
*anmt-1*^dopa^ rescues DA neurodegeneration in toxin-induced and genetic *C*. *elegans* models of PD. **a** DA neuronal morphology of wt and *anmt-1*^dopa^ categorized in no (white), slight (light grey), and severe (dark grey) treated with β-hexachlorocyclohexane (β-HCH; 1 mM) compared to DMSO, paraquat (PQ; 300 μM) and 6-hydroxydopamine (6-OHDA; 1mM) compared to water at 10 days of adulthood. See S1 Table for statistics. **b** Presence of (DA) cell bodies in *anmt-1*^dopa^ (grey), *SNCA-*A53T^dopa^ (dark green), *SNCA-*A53T^dopa^;*anmt-1*^dopa^ (green), *pdr-1(gk488)* (purple) and *pdr-1(gk488);anmt-1*^dopa^ (light purple) compared to wt (black) at day 15 of adulthood. **c** Abnormal DA cell body positioning at day 15 of adulthood. *: p < 0.05, **: p < 0.01, ***: p < 0.001.

After establishing a role of ANMT-1 in regulation of autophagy, especially in the neurons, and therefore preventing age-related neurodegeneration, we wondered if *anmt-1* expression was neuroprotective in disease-like states, induced by either PD-related neurotoxins or mutations. Thus, we tested a variety of compounds that have been associated with DA neurodegeneration and/or increased risk of PD onset to determine whether increased *anmt-1* expression influences the neurotoxicity of these compounds. Worms were exposed to the respective substances from the egg stage and DA morphology was measured at L4, day 5 and day 10 of adulthood. β-hexachlorocyclohexane (β-HCH) is classified as persistent organic pollutant and serum levels may correlate with PD onset risk [49]. We tested the ability of β-HCH (1 mM) to damage *C*. *elegans* DA neurons and found increased morphological damage in wt as early as in the L4 stage when compared to DMSO **(S7A and S7B Fig, S1 Table for statistics)**. Paraquat (PQ; 300 μM) and 6-OHDA (1 mM) caused a trend towards DA morphological damage at L4 and day 5 (**S7A, S7B, S7C and S7D Fig)** that became significant in 10-day old worms **(Figs 7A and S7E)** when compared to controls. Both compounds can damage DA neurons, which was reported previously in *C*. *elegans* [50, 51] and other organisms as well as humans [52–54], and are therefore suspected to cause PD. Surprisingly, *anmt-1*^dopa^ worms seemed to be completely protected against the neurotoxic effects of β-HCH, PQ, and 6-OHDA **(Figs 7A, S7A, S7B, S7C, S7D and S7E)**. While this neuroprotective effect of ANMT-1 may be due to increased autophagy, other mechanisms could contribute to this resilience, such as upregulated stress response caused by oxidative stress, which is evoked by PQ and 6-OHDA and has been reported previously for ANMT-1 [19]. However, given the structural and functional differences of the tested compounds, it is likely that the neuroprotection caused by *anmt-1*^dopa^ is a general effect rather than specific to a particular family of molecules.

To investigate whether *anmt-1* interacts with genetic risk factors for PD and influences their pathologies, we tested a variety of disease models. An autosomal-dominant mutation in the alpha-synuclein gene *SNCA* (an alanine-to-threonine substitution at position 53, A53T) was the first discovered to be responsible for heritable cases of PD [6, 55]. Shortly after, mutations in the parkin gene *PARK2* were found to be responsible for autosomal-recessive juvenile PD [7]. Later, mutations in both genes have been found to contribute to sporadic PD cases as well [56]. Therefore, we employed a *C*. *elegans* model that expresses human *SNCA* A53T in their DA neurons (P_*dat-1*_::*SNCA-*A53T; *SNCA-*A53T^dopa^), and *pdr-1(gk488)*, a loss of function mutant of *pdr-1*, the worm orthologue of human *PARK2*. As previously shown [57, 58], we found that both strains show degeneration of their DA neurons at day 5 of adulthood, as indicated by the loss of DA cell bodies **(S8A Fig)** and abnormal positioning of these cells **(S8B Fig)**, and other morphologic anomalies of the DA system **(S8C and S8D Fig)**. When *anmt-1* was expressed in the DA neurons of these strains (*SNCA-*A53T^dopa^;*anmt-1*^dopa^ and *pdr-1(gk488)*;*anmt-1*^dopa^), we found that the loss and abnormal positioning of DA cell bodies compared to wt was completely abolished at day 5 **(S8A and S8B Fig)** and day 15 **(Figs 7B, 7C and S8E)**, and dysmorphia of CEP dendrites **(S8C and S8F Fig)**, and axonal breaks **(S8D and S8G Fig)** are the same as in *anmt-1*^dopa^. SNCA tends to build aggregates, especially when mutated. It has furthermore been reported that SNCA and SNCA-A53T might diminish autophagic processes [59]. The E3 ubiquitin ligase PARK2 is an important mediator of mitophagy, which is the selective autophagic degradation of mitochondria. We speculate that the effects of *SNCA* A53T and *pdr-1* loss on DA neurons are, at least in part, due to accumulation of aggregated SNCA and damaged mitochondria, respectively. *anmt-1* expression induces autophagic processes, which could lead to reduction of these aggregates and dysfunctional organelles, restoring neuronal function.

## Discussion

**Fig 8:**
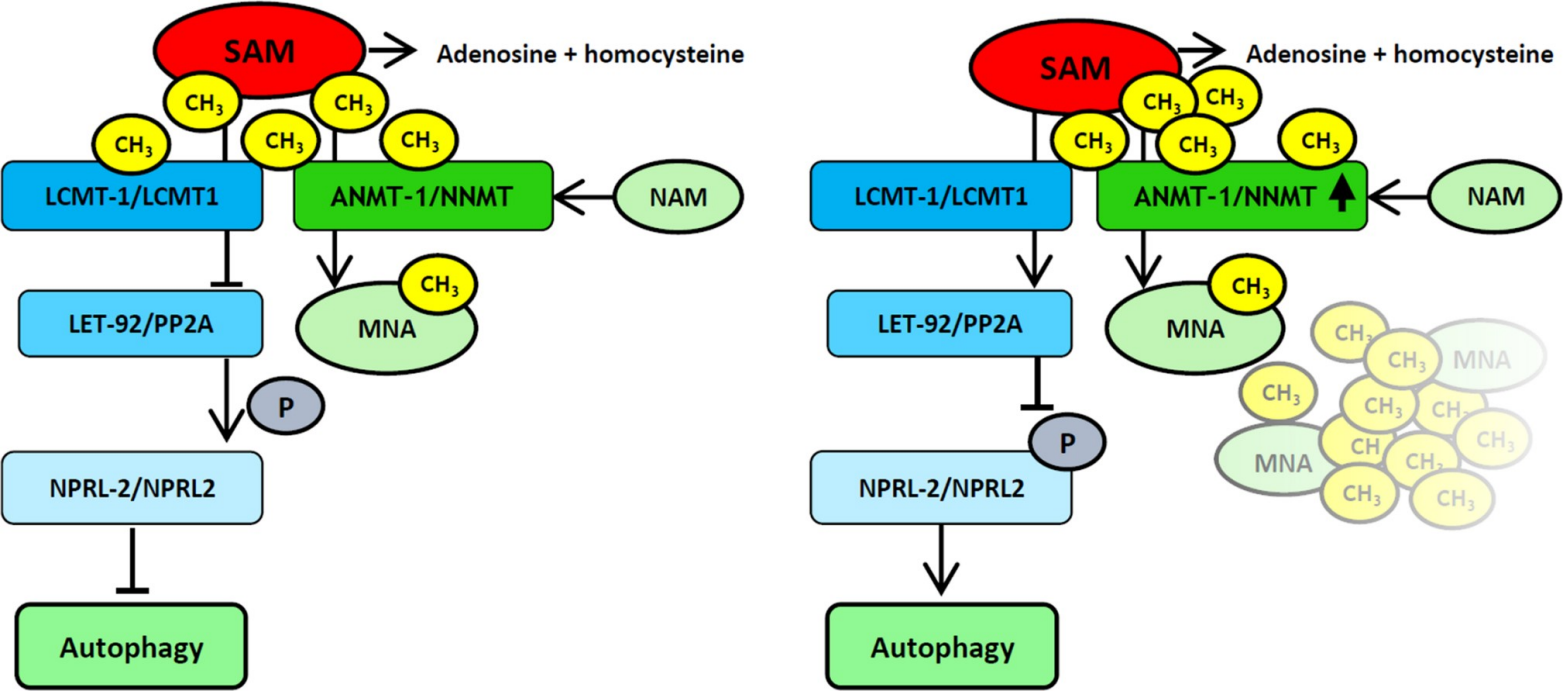
Graphic summary. ANMT-1/NNMT methylates nicotinamide (NAM) to N-methylnicotinamide (MNA), using S-adenosyl methionine (SAM) as methyl group donor. The reaction is irreversible, *i*.*e*. methyl groups are lost for metabolism and SAM cannot be recycled. This reduces cellular SAM concentration, thus reducing the catalyzing capability of the methyltransferase LCMT-1/LCMT1 (right). Under normal cellular SAM levels (left), LCMT-1/LCMT1 methylates the phosphatase LET-92/PP2A in order to activate it. Active LET-92/PP2A would dephosphorylate NPRL-2/NPRL2, leading to inhibition of autophagy. Unmethylated, therefore inactive LET-92/PP2A (right) cannot dephosphorylate NPRL-2/NPRL2, which will induce autophagy.

We explored the neuronal role of ANMT-1/NNMT *in vivo* and found that it regulates neuronal autophagy (**Fig 8**) in the DA nervous system, with wide-ranging effects on neurodegeneration, behavior, fertility, and lifespan.

NNMT has been previously reported as eliciting contradictory outcomes regarding PD risk: elevated NNMT levels were found in the brains and lumbar cerebrospinal fluid of PD patients [28, 60, 61], whereas other studies in cell tissue culture found it to be neuroprotective [32, 62–64]. Our data suggest a neuroprotective role for ANMT-1/NNMT, but it cannot be ruled out that higher expression levels and/or encountering other risk factors could lead to further dysregulation and neurodegeneration. The neuroprotection results from the deprivation of SAM, which likely acts as starvation signal to the cell. It has been shown that SAM reciprocally regulates autophagy, promoting growth under high concentrations and boosting autophagy when levels decrease [37, 38]. SAMS-1, the key enzyme in SAM biosynthesis, was initially identified in an RNAi screen for positive regulators of longevity via dietary restriction [43]. *sams-1* mutants show extended lifespan and mimic other phenotypes of DR worms, such as reduced brood size and delayed reproduction [65], resembling the phenotypes that we observe in *anmt-1*^dopa^ worms. Reduced SAMS-1 mRNA levels as in *anmt-1*^dopa^ have also been described in genetic models of dietary restriction [43]. We therefore hypothesize that high neuronal ANMT-1/NNMT activity mimics dietary restriction by reducing the availability of cellular SAM, leading to lifespan extension. Furthermore, decreased SAM, and hence reduced methylation potential, could modulate histone and DNA methylation and affect epigenetic processes [22].

In young (5 day old) *anmt-1*^dopa^ individuals, however, dopamine-dependent behavior is disturbed, which could be due to increased dopamine levels in *anmt-1*^dopa^ animals. It is interesting to note that schizophrenia, with a general onset age in early adulthood [66], is associated with excessive dopamine, leading to abnormal signaling and the typical behavioral outcomes. Therapy involves the use of antipsychotic drugs that block dopamine receptors, whereas drugs that drive dopamine release or increase dopamine transmission, such as amphetamines, will exacerbate psychosis in patients with schizophrenia, and can induce schizophrenia-like symptoms in otherwise healthy individuals [15]. NNMT has been associated with schizophrenia in humans [23–25], which according to our results may be due to its influence on dopamine concentration and/or signaling. Furthermore, autophagy dysregulation in the brain plays a key role in the pathology of schizophrenia [13], and an NNMT-mediated increase in autophagy could therefore also contribute to the progression of the disease. Since ANMT-1/NNMT seems to increase autophagy levels independently of age, perhaps levels are too high in young adulthood, and become beneficial only with age as the incidence of damaged macromolecules and dysfunctional organelles in the neurons increase. Increased autophagic clearance could therefore be the basis of the ANMT-1/NNMT-dependent neuroprotection and lifespan extension we observed. Autophagy has been linked to longevity in many organisms and an emerging field of investigation concerns the differential regulation of autophagy during aging, and effects on longevity [67] and neurodegeneration [68] have been reported depending on the relative age of the organism in question. We chose to examine neurodegeneration phenotypes at the age of 15 days after L4, which may resemble the human age that is most prevalent for PD onset (around 65 years) [69]. Future research could continue in this direction and establish whether an increase in autophagic processes only in older age is sufficient to mediate the beneficial effects without influencing other autophagy-sensitive diseases in younger individuals. Notably, we also found some neurodevelopmental issues in L4 larvae (S1E Fig) that were expected to not experience any neuronal loss, which could not be confirmed by our analysis. It would be interesting to further investigate whether the individuals that have neurodevelopmental problems experience an earlier onset or faster progression of neurodegeneration in older age.

Given the influence of *anmt-1* expression in the DA neurons on dopamine, and the dependency of lifespan extension on dopamine production, we speculate that dopamine might act beyond its known functions and perhaps via receptors not yet described.

However, we found a greater loss of DA cells and higher morphological damage, *i*.*e*. features of PD, in *anmt-1*^dopa^ than in wt animals when autophagy was abrogated. Maybe this secondary increase in neurodegeneration, when ANMT-1/NNMT levels are high and autophagy is dysfunctional, could account for the increased NNMT expression in PD patients observed in other studies [28, 60].

The LCMT-1/NPRL-2 pathway, which links ANMT-1/NNMT to autophagy regulation, also involves PP2A. We were not able to test the worm ortholog LET-92, however, given its high expression levels in the nervous system, it is likely to have an important neuronal role [46]. Recently, it has been reported that NNMT silencing is able to activate PP2A via its effects on LCMT-1 in glioblastoma cells [70]. Subsequently, this activation of PP2A lead to inactivation of serine threonine kinases (STKs). A genome-wide association study on PD in large populations of Europe and the USA found that polymorphisms in the gene encoding STK39 significantly increases the risk for PD [10]. Thus, in the context of dysfunctional autophagy, NNMT might modulate the activity of STKs such as STK39 to trigger DA neurodegeneration, while under wt conditions the overall beneficial effects of autophagy outweigh the potentially damaging effect of modulating STK39. Interestingly, the antipsychotic drug perphenazine, currently used to treat schizophrenia, activates PP2A and rescues a potential PP2A inhibition by NNMT [70].

Taken together, our research shows the contribution of NNMT to neuroprotection and its involvement in neuronal diseases and provides evidence for autophagy as underlying biochemical pathway. We have detailed this novel molecular mechanism regulating neuronal autophagy during aging and raise the possibility of the NNMT pathway as a potential target for neuroprotective interventions in PD, schizophrenia, and other neurological diseases. Further research is required to enlighten the DA-neuronal specificity of NNMT action, and to investigate how epigenetic regulation intervenes in these processes.

## Methods

### *C*. *elegans* maintenance and strains

*C*. *elegans* were maintained as described elsewhere [71]. Briefly, worms were kept on NGM agar plates that were streaked with *E*. *coli* OP50 as food source at 15°C. All assays were performed at 20°C, and worms were grown at 20°C at least one generation before the experiment. The N2 Bristol wildtype strain (wt), as well as BZ555 (P_*dat-1*_::GFP; GFP^dopa^), MT15620 (*cat-2(n4547)*), LX703 (*dop-3(vs106)*), VK1093 (*vkEx1093*[*nhx-2*p::mCherry::*lgg-1*]), TU3401 (*sid-1(pk3321)*;[pCFJ90 (P_*myo-2*_::*mCherry*) + *unc-119p*::*sid-1*]), RB2240 (*sams-1(ok3033)*), and VC1024 (*pdr-1(gk488)*) were provided by the Caenorhabditis Genetics Center at the University of Minnesota. Mutant strains were outcrossed to wt at least 4 times. Strains MIR8 (P_*anmt-1*_::*anmt-1*::GFP; *anmt-1*^OEx^) and MIR16 (*anmt-1(gk457)*) were made as described previously [19]. FX14471 (tmIS904; P_*dat-1*_::*a-syn* A53T) were generated by the Iwatsubo lab [72] and obtained from Dr. Shohei Mitani. Other *C*. *elegans* strains obtained by crossing and used in this study can be found in Table 2. Homozygosity of all genotypes was confirmed by PCR.

**Table 2:** Newly generated *C*. *elegans* strains.

Strain name	Original strains	genotype
XQ608		P_*dat-1*_* *::*anmt-1* (anmt-*1*^dopa^)
XQ609		P_*unc-47*_* *::*anmt-1* (*anmt-1*^GABA^)
XQ610	XQ608;BZ555	*anmt-1*^dopa^;P_*dat-1*_::GFP
XQ611	XQ609	*anmt-1*^GABA^;P_*unc-47*_::mCherry
XQ612	XQ608;MT15620	*anmt-1*^dopa^;*cat-2(n4547)*
XQ613	XQ608;LX703	*anmt-1*^dopa^;*dop-3(vs106)*
XQ614	RB2240;BZ555	*sams-1(ok3033)*;P_*dat-1*_::GFP
XQ615	XQ608;RB2240;BZ555	*anmt-1*^dopa^;*sams-1(ok3033)*;P_*dat-1*_::GFP
XQ616	TU3410;BZ555	(*sid-1(pk3321)*;[pCFJ90 (P_*myo-2*_::*mCherry*) + *unc-119p*::*sid-1*]);P_*dat-1*_::GFP
XQ619	XQ608;TU3410;BZ555	*anmt-1*^dopa^;(*sid-1(pk3321)*;[pCFJ90(P_*myo2*_::*mCherry*) + *unc-119p*::*sid-1*]);P_*dat-1*_::GFP
XQ620	MIR8;VK1093	*anmt-1(gk457)*;vkEx1093 [*nhx-2*p::mCherry::*lgg-1*]
XQ621	MIR16;VK1093	*anmt-1*^dopa^;vkEx1093 [*nhx-2*p::mCherry::*lgg-1*]
XQ622	RB2240;VK1093	*sams-1(ok3033)*;vkEx1093 [*nhx-2*p::mCherry::*lgg-1*]
XQ623	MIR8;RB2240;VK1093	P_*anmt-1*_::*anmt-1*;*sams-1(ok3033)*; vkEx1093 [*nhx-2*p::mCherry::*lgg-1*]
XQ624	MIR16;RB2240;VK1093	*anmt-1(gk457)*;*sams-1(ok3033)*; vkEx1093 [*nhx-2*p::mCherry::*lgg-1*]
XQ625	FX14471;BZ555	TmIS904 [P_*dat-1*_::*a-syn A53T*];P_*dat-1*_::GFP
XQ626	XQ608;FX14471;BZ555	*anmt-1*^dopa^;TmIS904 [P_*dat-1*_::*a-syn A53T*];P_*dat-1*_::GFP
XQ627	VC1024;BZ555	*pdr-1(gk488)*;P_*dat-1*_::GFP
XQ628	XQ608;VC1024;BZ555	*anmt-1*^dopa^;*pdr-1(gk488)*;P_*dat-1*_::GFP
XQ630	MIR8;AM140	P_*anmt-1*_::*anmt-1*;rmIs132[P_*unc-54*_::*Q35*::*YFP*]
XQ631	MIR16;AM140	*anmt-1(gk457)*;rmIs132[P_*unc-54*_::*Q35*::*YFP*]
XQ632	MIR8;GF80	P_*anmt-1*_::*anmt-1*; dgEx80[(pAMS66);P_*vha-6*_::*Q44*::*YFP* + *rol-6(su1006)* + pBluescript II]
XQ633	MIR16;GF80	*anmt-1(gk457)*; dgEx80[(pAMS66);P_*vha-6*_::*Q44*::*YFP* + *rol-6(su1006)* + pBluescript II]
XQ634	MT15620;BZ555	*cat-2(n4547)*;P_*dat-1*_::GFP
XQ635	LX703;BZ555	*dop-3(vs106)*;P_*dat-1*_::GFP
XQ636	XQ608;MT15620;BZ555	*anmt-1*^dopa^;*cat-2(n4547)*;P_*dat-1*_::GFP
XQ637	XQ608;LX703;BZ555	*anmt-1*^dopa^;*dop-3(vs106)*;P_*dat-1*_::GFP
XQ638		P_*dat-1*_* *::*anmt-1*(Y35A;Y40A;Y80A;D96A;N101A;D219A) (*anmt-1*^dopa-MUT 1^), extrachromosomal
XQ639		P_*dat-1*_* *::*anmt-1*(Y35A;Y40A;Y80A;D96A;N101A;D219A) (*anmt-1*^dopa-MUT 2^), extrachromosomal

Transgenic animals were generated as follows. Plasmid DNA with the *anmt-1* gene under the control of either a DA-neuronal (*dat-1*) or GABA-motor neuron specific promoter (*unc-47*) was prepared using the Gateway Cloning system and the site-specific vector pCFJ606. Transgenics were generated by microinjection of plasmid DNA and stably integrated into a defined site of the genome (locus *ttTi14024*, position X:22.84) using the MosSCI technique. Alternatively, the *dat-1*p::*anmt-1* construct, or a mutated version of this construct, together with a co-injection marker were injected into BZ555, resulting in extrachromosomal expression of either wt or mutated *anmt-1* in the DA nervous system. *anmt-1* was mutated using site-directed mutagenesis, and two resulting lines were analyzed (*anmt-1*^dopa-MUT 1^ and *anmt-1*^dopa-MUT 2^). Strains were outcrossed 4 times to wt.

### Site-directed mutagenesis of *anmt-1*

Amino acid residues of ANMT-1 that are important for SAM binding were identified using UniProt [73] (entry P34254), which provided 5 potential binding sites at positions 35 (tyrosine; Y), 40 (Y), 80 (Y), 96 (aspartic acid; D) and 101 (asparagine; N). Protein sequence alignment of *C*. *elegans* ANMT-1 and human NNMT showed high conservation between these residues. Analysing the crystal structure of human NNMT, Peng *et al*. reported an additional important residue at position 197 (D) [74], which is potentially conserved with a small gap, given the existence of a D in the ANMT-1 sequence at position 219 that matches the amino acid context of D197 in NNMT. All identified potentially active residues where replaced by alanine (A), resulting in the following mutations: Y35A, Y40A, Y80A, D96A, N101A, and D219A. Mutations were generated using a QuikChange II XL Site-Directed Mutagenesis Kit (Agilent Technologies).

### RNAi experiments and bacterial strains

RNAi experiments were performed according to Kamath *et al*. [75]. RNAi clones (*E*. *coli* HT115) of *bec-1*(T19E7.3), *atg-13*(D2007.5), *lgg-1*(C32D5.9), *lcmt-1*(B0285.4) and *nprl-2*(F49E8.1) were taken from the ORFeome RNAi library (Open Biosystems) and compared to an empty vector clone (L4440). Sequencing to confirm correct clones was performed for all RNAis before use. Adult worms were put on RNAi NGM plates containing 1  mM isopropyl-ß-D-thiogalactopyranoside and 50 μg/ml ampicillin and allowed to lay eggs for about 4 hours. Progeny from L4 on was transferred every two days to avoid contamination with younger generations. Neuronal RNAi experiments were performed using the respective strain crossed into TU3401 for neuronal-specific gene silencing.

### Neuronal fluorescence microscopy

Synchronized worms of different ages were placed on microscopy slides with 2% agarose pads and immobilized with 5 mM levamisole in M9 buffer. Neuronal fluorescence microscopy was conducted with a Zeiss Axio Imager M2 microscope and Zen Pro software (Carl Zeiss Canada) with 40x amplification. Present DA and GABA cell bodies and GABA commissures were counted, and worms were screened for breaks in axons and dysmorphia (breaks, punctated GFP signal, dislocation) in CEP dendrites, and abnormal DA cellular positioning. At least fifteen animals were analyzed in each of at least 3 independent experiments per condition and two-tailed t-test was performed to determine significance.

### Fertility assay

Single hermaphrodites at the L4 stage were put on NGM plates and allow to lay eggs. Parental worms were transferred to fresh plates every 12 hours for the first 3 days, then every 24 hours until they stopped laying eggs. Progeny that reached L4 per parental worm was counted. Three independent experiments were performed in quadruplicates.

### Lifespan assay

Lifespan assays were performed as previously described [19]. Briefly, worms were synchronized at the egg stage (day 0). At L4, around 50 nematodes were transferred to each of 3 fresh lifespan plates per condition. After 24–48 hours, worms were transferred on plates containing 10 μM FUDR to prevent progeny contamination. FUDR was solved in water and applied on top of the grown bacteria lawn. *C*. *elegans* that did not react to repeated gentle stimulation were scored as dead. Lost animals or non-natural deaths (bagging, protrusive vulvae) were censored. JMP 11.0.0 (SAS institute Inc.) was used for statistical analyses (see Table 3).

**Table 3:** Results and statistical analyses of lifespan assays.

Genotype, Strain, RNAi	Maximum lifespan [d] +/- SEM^1^	Mean lifespan [d] +/- SEM	p-value (vs. Control^2^)	Number of experiments [n]	Number of nematodes [n]
**wt (N2)**	27.6 +/-0.5	18.54 +/-0.2		5	444
***anmt-1***^**dopa**^ **(XQ608)**	30.8 +/-19	21.08 +/-0.2	<0.0001 ^a^	5	486
***anmt-1***^**dopa-MUT**^ ^**1**^ **(XQ638)**	27.0 +/-2.0	19.67 +/-0.5	<0.0001 ^b^	2	78
***anmt-1***^**dopa-MUT**^ ^**2**^ **(XQ639)**	27.7 +/-1.2	19.01 +/-0.4	<0.0001 ^b^	2	89
***cat-2*(*n4547)* (MT15620)**	28.5 +/-0.8	19.21 +/-0.2	0.0209 ^a^	3	257
***cat-2*(*n4547*);*anmt-1***^**dopa**^ **(XQ612)**	28.0 +/-2.5	18.97 +/-0.2	n.s. ^c^	3	388
***dop-3(vs106) (LX703)***	26.5 +/-2.1	17.66 +/-0.2	n.s. ^a^	3	222
***dop-3(vs106*);*anmt-1***^**dopa**^ **(XQ613)**	29.3 +/-2.6	19.26 +/-0.2	<0.0001 ^d^	3	336
***anmt-1***^**GABA**^ **(XQ209)**	28.9 +/-1.9	20.20 +/-0.2	<0.0001 ^a^	4	417
***sams-1(ok3033*) (RB2240)**	29.0 +/-0.0	20.43 +/-0.2	<0.0001 ^a^	3	220
***sams-1(ok3033*);*anmt-1***^**dopa**^ **(XQ614)**	27.5 +/-0.7	20.03 +/-0.2	n.s.^e^	3	272
***sid-1(pk3321);*P**_***unc-119***_**::*sid-1* (TU3401) (EV RNAi)**	28.8 +/-1.0	19.98 +/-0.2		7	703
***sid-1(pk3321);*P**_***unc-119***_**::*sid-1* (TU3401) (*atg-13* RNAi)**	32.0 +/-1.4	21.16 +/-0.2	<0.0001 ^f^	3	285
***sid-1(pk3321);*P**_***unc-119***_**::*sid-1* (TU3401) (*bec-1* RNAi)**	31.0 +/-0.0	20.85 +/-0.3	0.0002 ^f^	3	299
***sid-1(pk3321);*P**_***unc-119***_**::*sid-1* (TU3401) (*lgg-1* RNAi)**	25.0.+/-1.4	17.97 +/-0.2	<0.0001 ^f^	3	191
***sid-1(pk3321);*P**_***unc-119***_**::*sid-1* (TU3401) (*lcmt-1* RNAi)**	30.7 +/-2.3	19.78 +/-0.3	n.s.^f^	3	211
***sid-1(pk3321);*P**_***unc-119***_**::*sid-1* (TU3401) (*nprl-2* RNAi)**	31.0 +/-1.7	21.22 +/-0.2	<0.0001 ^f^	3	344
***sid-1(pk3321);*P**_***unc-119***_**::*sid-1*;*anmt-1***^**dopa**^ **(XQ619) (EV RNAi)**	30.7 +/-0.6	20.75 +/-0.2	<0.0001 ^f^	7	701
***sid-1(pk3321);*P**_***unc-119***_**::*sid-1*;*anmt-1***^**dopa**^ **(XQ619) (*atg-13* RNAi)**	23.0 +/-0.0	16.09 +/-0.2	<0.0001 ^g^	2	181
***sid-1(pk3321);*P**_***unc-119***_**::*sid-1*;*anmt-1***^**dopa**^ **(XQ619) (*bec-1* RNAi)**	23.5 +/-0.7	16.99 +/-0.2	<0.0001 ^g^	4	404
***sid-1(pk3321);*P**_***unc-119***_**::*sid-1*;*anmt-1***^**dopa**^ **(XQ619) (*lgg-1* RNAi)**	22.0 +/-2.8	15.75 +/-0.1	<0.0001 ^g^	3	320
***sid-1(pk3321);*P**_***unc-119***_**::*sid-1*;*anmt-1***^**dopa**^ **(XQ619) (*lcmt-1* RNAi)**	27.0 +/-1.4	18.99 +/-0.2	0.0002 ^g^	3	338
***sid-1(pk3321);*P**_***unc-119***_**::*sid-1*;*anmt-1***^**dopa**^ **(XQ619) (*nprl-2* RNAi)**	28.0 +/-0.0	19.34 +/-0.1	0.0005 ^g^	4	455
***sid-1(pk3321);*P**_***unc-119***_**::*sid-1*;*anmt-1***^**dopa**^ **(XQ619) (*anmt-1* RNAi)**	23.7 +/-1.2	17.59 +/- 0.2	<0.0001 ^g^	3	191

^1^ day last individual died

^2^controls

^a^ wt (N2)

^b^ *anmt-1*^dopa^ (XQ608)

^c^ *cat-2*(*n4547)* (MT15620)

^d^ *dop-3*(*vs106*) (LX703)

^e^ *sams-1(ok3033*) (RB2240)

^f^ *sid-1(pk3321);*P_*unc-119*_::*sid-1* (TU3401) (EV RNAi)

^g^ *sid-1(pk3321);*P_*unc-119*_::*sid-1*;*anmt-1*^dopa^ (EV RNAi)

### Basal slowing response assay

The assay plates were prepared as follows: an about 1 cm diameter droplet of OP50 was placed on one site of a 10 cm NGM petri dish and allowed to dry. About 100 well fed worms per experiment were placed on an empty NGM plate and let crawl up to an hour to get rid of excessive bacteria. Worms were then transferred to assay plates on the opposite site of the bacterial lawn and allowed to move for 1 hour. Subsequently, worms inside and outside the bacterial lawn were counted and the basal slowing index was calculated as follows: (worms outside of lawn)-(worms in lawn)/(complete number of worms), where a result between 1 and 0 represents a healthy behavior.

For verification we used an alternative method of testing basal slowing according to [35].Worms were synchronized at the L4 stage and experiments were performed at day 1, 5, and 10 of adulthood. 35 μl of an overnight *E*. *coli* OP50 culture were spread on a 6 cm agar dish and incubated over night at 37°C. Control plates without bacteria were treated the same. Animals were washed free of bacteria with M9. After 3 min in M9, worms were allowed a 90 sec recovery period on the respective assay plate. Subsequently, body bends were counted for five consecutive 20 sec periods. A body bend was defined as a change of direction of the complete head and pharynx region relative to the vertical axis. At least 5 animals per condition were tested.

### Ethanol avoidance assay

The assay plates were prepared as follows: a 10 cm NGM plate streaked with OP50 was quartered. 60 μl 96% ethanol was pipetted on two quarters of the plate and allowed to dry for about 5 mins. About 100 well fed worms per experiment were placed in the center of the plate. Worms were allowed to move freely for 1 hour. Subsequently, worms inside and outside the ethanol quadrants were counted and the chemotaxis index was calculated as follows: ((worms outside of lawn)-(worms in lawn)/(complete number of worms))*-1, where a result between 1 and 0 represents a healthy behavior.

### Dopamine pre-treatment

Dopamine drug pre-treatment was performed as described previously [36]. In sum, a 50 mM dopamine hydrochloride solution in M9 buffer was prepared freshly before the assay. 400 μl of this solution were put on a 5 cm NGM plate seeded with OP50 and allowed to dry. For control, 400 μl M9 was added. Worms were put on the prepared dopamine and control plates 4 to 6 hours before the basal slowing response and ethanol avoidance assay.

### Locomotion

In a 96-well-plate, 30 age-synchronized worms were transferred into a well filled with 100 μl M9 buffer and OP50. Swimming locomotion was automatically tracked for 10 h using a worm tracking machine (Wmicrotracker, Phylum Tech) that performs measurements as follows. Each microtiter well is crossed by two infrared light rays from top to bottom. A detector determines how often the light rays were interrupted by worms moving in the well, and the signal is used to calculate a movement score, which is the amount of animal movement in a fixed time period. All measurements were performed in triplicates in 3 independent experiments and compared to wt worms of the same age.

### Autophagy assay/puncta formation

Worms were synchronized, grown on OP50 or RNAi bacteria from the egg stage and transferred to fresh plates every 2 days from day 1 of adulthood. Compound plates (positive/negative control plates) were poured fresh before each assay, with pimozide at a concentration of 20 μM and 3-methyladenine at 5 mM dissolved in DMSO. Assays were performed at different ages as follows. Worms were put on empty streptomycin plates for about an hour to get rid of excess bacteria. About 24 hours before the assay, fed worms went back on fresh plates with food, starved worms were placed on the same plates that were not streaked with bacteria. For RNAi experiments, the starvation period was about 16 hours. For puncta assessment, worms were put on microscope slides with 2% agarose pads and immobilized with levamisole, and assessment was performed with Zeiss Axio Imager M2 microscope and Zen Pro software at 587 nm excitation/610 nm emission for mCherry. Pictures were taken and analysed with the “Find maxima” function of ImageJ 1.49V. Heterozygous strains (used when homozygosity of genotype caused sterility) were put in lysis buffer immediately after microscopy and stored at -20°C for single worm PCR to determine genotype.

### HPLC ESI-MS/MS analysis

Detection of dopamine, GABA, SAM and one-carbon metabolites was performed via HPLC (high performance liquid chromatography) coupled with ESI-MS/MS (electrospray ionisation tandem mass spectrometry) detection. The method was adapted from Wojnicz et al. [76]. Metabolites were extracted by sonication in acidified water (1.89% formic acid; sonication in ice-cold water for a total of 40 sec, with pulses of 10 sec at 40% intensity using a micro tip probe) followed by acetonitrile protein precipitation and sample concentration by drying using a refrigerated CentriVap set at 10°C. Reconstituted samples kept at 4°C were injected (30μL) and separated by a Nexera X2 HPLC system (Shimadzu) using a C18-PFP column 4.6 x 150 mm, 3 μm particle size (ACE, Scotland) protected by a C18-PFP guard column 3.0 x 10mm, 3μm particle size (ACE, Scotland); column compartment set at 30°C; gradient elution at 0.6mL/min in mobile phases A (0.1% formic acid in H_2_Odd) and B (acetonitrile) as follows: 0 min 5% B, 2 min 5% B, 5 min 90% B, 8 min 90% B, 10 min 5% B, 14 min 5% B. Detection was performed by ESI-MS/MS in positive ion mode on a 6500 QTrap (Sciex). Transitions used were for GABA 104.0 = >87.0 (collision energy (CE):15), for d2-GABA 106.0 = >89.0 (CE:15), for dopamine 154.0 = >91.0 (CE:33), for d3-dopamine 157.0 = >93.0 (CE:46), for SAM 399.0 = >250.1 (CE:21), for d3-SAM 402.0 = >250.0 (CE:25), and 136.0 = >90.0 (CE:15) for homocysteine.

### Gene expression analysis

Total RNA was obtained using Trizol (Invitrogen)/chloroform extraction as described previously [19], quantified photometrically with a NanoPhotometer (Implen) and stored at -80°C until further use. cDNA from 500 ng total RNA was generated using QuantiTect reverse transcriptase (Qiagen) and diluted 1:10 to 1:1000 to determine a concentration for each gene that yielded a CT value between 15 and 25. Gene expression was analyzed using TaqMan Gene Expression Assays (Applied Biosystems) and a QuantStudio 3 Real-Time PCR System (Thermo Fisher). Data were normalized to the housekeeping gene *cdc-42* and analyzed using the Δ/Δ-CT method.

### Neurotoxicity assay

Compound plates were poured fresh before each assay and streaked with OP50. We chose a concentration of each compound where there was no visible impairment of bacterial or nematode growth. Beta-chlorocyclohexane (β-HCH) was dissolved in DMSO and used at a concentration of 1 mM. Paraquat (PQ) and 6-Hydroxydopamine (6-OHDA) were dissolved in water and tested at concentrations of 300 μM and 1 mM, respectively. All compounds were obtained from Sigma. Young adult worms were allowed to lay eggs on compound plates for about 4 hours. Progeny was investigated via neuronal fluorescence microscopy at L4, day 5, and day 10, before a significant proportion of the population started to die. Worms were transferred every 2 days to fresh plates.

Morphology of the DA system was calculated from cell body count and positioning, presence of axonal breaks, and dysmorphia in CEP dendrites. The category of no degeneration (“none”) was assumed when average cell body presence was > 95%, and < 20% of animals showed axonal breaks and abnormal positioning, abnormal cell body positioning, and dysmorphia in CEP dendrites. Slight or severe degeneration was assumed when average cell body presence was > 50% or < 50%, and axonal breaks, abnormal cell body and axon positioning, and dysmorphia in CEP dendrites occurred in < 60% and > 60% of animals, respectively. At least fifteen animals were analyzed in each of at least 3 independent experiments per condition and two-tailed t-test was performed to determine significance.

## Supporting information

S1 Fig *anmt-1*^dopa^ influences degeneration rather than development. **a** Representative pictures of GFP^dopa^ in L4 and young adults with no, slight, and severe degeneration of the DA neuronal system. Cell bodies, axons, and dendrites are marked with arrows, and pathological features are marked with arrowheads. Colored letters A and P indicate anterior and posterior, respectively. Orientation is the same in all panels. **b** Presence of CEP, ADE, and PDE cell bodies in wt (black) and *anmt-1*^dopa^ (grey) at day 15 of adulthood. **c** Presence of CEP, ADE, and PDE cell bodies in wt (black) and *anmt-1*^dopa^ (grey) at day 5 of adulthood. **d** DA neuronal morphology categorized in CEP dendrite dysmorphia, axonal breaks, and abnormal cell body and axon positioning in percentage of individuals of wt and *anmt-1*^dopa^ at day 5 of adulthood. **e** Presence of DA cell bodies in wt and *anmt-1*^dopa^ at L4. **f** Presence of DA cell bodies in two *anmt-1*^dopa–MUT^ lines (purple, light purple) compared to wt and *anmt-1*^dopa^ at day 15 of adulthood. **g** Presence of DA cell bodies in wt (black) and *anmt-1*^dopa^ (grey) treated with empty vector RNAi (EV) and *anmt-1*^dopa^ treated with *anmt-1* RNAi from the egg stage (green) and from the L4/day 1 stage (light green) at day 15 of adulthood. * compared to wt EV; # compared to *anmt-1*^dopa^ EV. **h** Lifespan analysis of two *anmt-1*^dopa–MUT^ lines compared to wt and *anmt-1*^dopa^. **i** Lifespan analysis of two *anmt-1*^dopa^ treated with RNAi against *anmt-1* (light blue) compared to EV (grey). **j** Number of L4 progeny in *anmt-1*^dopa^ compared to wt. **k** Presence of CEP, ADE, and PDE cell bodies in wt treated with 1 μM MNA (black) compared to water as control (Ctrl; white) at day 15 of adulthood. **l** DA neuronal morphology categorized in CEP dendrite dysmorphia and axonal breaks in wt treated with 1 μM MNA compared to water as control at day 15 of adulthood. *: p < 0.05, **^/##^: p < 0.01, ***^/###^: p < 0.001 f and g * compared to wt, ^#^ compared to *anmt-1*^dopa^
(TIF)

S2 Fig *anmt-1* expression in the GABAergic neuronal system shows less effects than in the DA neuronal system. **a** Representative pictures of L4 *anmt-1*^GABA^ marked with mCherry^GABA^. GABAergic neuronal cell bodies are marked with arrows, and commissures and axons are marked with arrowheads. **b** GABAergic cell bodies in wt (black) and *anmt-1*^GABA^ (grey) at day 15 of adulthood. **c** GABAergic commissures in wt and *anmt-1*^GABA^ at day 15 of adulthood. **d** Presence of GABAergic axonal breaks in wt and *anmt-1*^GABA^ at day 15 of adulthood in percentage of healthy individuals. **e** Lifespan analysis of *anmt-1*^GABA^ compared to wt. **f** Number of L4 progeny in *anmt-1*^GABA^ compared to wt. **g** Locomotion over a period of 10 h of *anmt-1*^GABA^ compared to wt at day 5 of adulthood. **h** Locomotion over a period of 10 h of *anmt-1*^GABA^ compared to wt at day 10 of adulthood. **i** GABA concentration in nmol/mg protein of a mixed population of wt and *anmt-1*^GABA^. ***: p < 0.001.
(PDF)

S3 Fig Neuronal autophagy mediates *anmt-1*^dopa^ induced phenotypes. **a–c** Basal slowing response tested as quantity of body bends per animal per 20 sec in wt and *anmt-1*^dopa^ animals on plates with (black) or without bacteria (white) at **a** day 1, **b** day 5, and **c** day 10 of adulthood. **d** Quantity of body bends per animal per 20 sec in two *anmt-1*^dopa-MUT^ lines on plates with or without bacteria at day 5 of adulthood. **e and f** Dopamine-dependent behaviors in wt, wt after 4–6 hours 50 mM dopamine pre-treatment (wt + dopamine; grey), *anmt-1*^dopa^, and *anmt-1*^dopa^ after 4–6 hours 50 mM dopamine pre-treatment (*anmt-1*^dopa^ + dopamine; rose) at day 5 of adulthood. **e** Basal slowing index. **f** Chemotaxis index. **g** Lifespan of *anmt-1*^dopa^;*cat-2(n4547)* (grey) compared to *cat-2(n4547)* (black). **h** Lifespan of *dop-3(vs106)*;*anmt-1*^dopa^ (grey) compared to *dop-3(vs106)* (black). **i** Presence of CEP, ADE, and PDE cell bodies in *cat-2(n4547);anmt-1*^dopa^ (light turquoise) compared to *cat-2(n4547)* (turquoise), and *dop-3(vs106)*;*anmt-1*^dopa^ (light pink) compared to *dop-3(vs106)* (pink) at day 15 of adulthood. *: p < 0.05, ** and ^##^: p < 0.01, ***: p < 0.001, e * compared to *cat-2(n4547)*, ^#^ compared to *dop-3(vs106)*.
(PDF)

S4 Fig Neuronal autophagy mediates *anmt-1*^dopa^ induced phenotypes. **a** Presence of CEP, ADE, and PDE cell bodies in *anmt-1*^dopa^ with a neuronal RNAi-sensitive background treated with RNAi against *bec-1* (orange), *atg-13* (green), or *lgg-1* (purple) compared to control RNAi (EV; black) and wt EV (patterned) at day 15 of adulthood. **b** Basal slowing index of wt treated with RNAi against *bec-1*, *atg-13* or *lgg-1* compared to EV. **c** Presence of CEP, ADE, and PDE cell bodies in wt with a neuronal RNAi-sensitive background treated with RNAi against *bec-1*, *atg-13*, or *lgg-1* compared to EV at day 15 of adulthood. **d** DA neuronal morphology in wt with a neuronal RNAi-sensitive background treated with RNAi against *bec-1*, *atg-13*, or *lgg-1* compared to EV at day 15 of adulthood. **e** Presence of CEP, ADE, and PDE cell bodies in *anmt-1*^dopa^ with a neuronal RNAi-sensitive background treated with RNAi against *bec-1*, *atg-13*, or *lgg-1* compared to EV and wt EV at day 5 of adulthood. **f** DA neuronal morphology in *anmt-1*^dopa^ with a neuronal RNAi-sensitive background treated with RNAi against *bec-1*, *atg-13*, or *lgg-1* compared to EV and wt EV at day 5 of adulthood. **g** DA neuronal morphology in wt with a neuronal RNAi-sensitive background treated with RNAi against *bec-1*, *atg-13*, or *lgg-1* compared to EV at day 5. **h** DA neuronal morphology in wt with a neuronal RNAi-sensitive background treated with RNAi against *bec-1*, *atg-13*, or *lgg-1* compared to EV at day 5 of adulthood. **i** Lifespan of wt with a neuronal RNAi-sensitive background treated with RNAi against *bec-1*, *atg-13* and *lgg-1* compared to EV. *: p < 0.05, **: p < 0.01, ***: p < 0.001.
(PDF)

S5 Fig ANMT-1 regulates autophagy by controlling SAM levels. **a** Number of puncta per individual worm in P_*nhx-2*_::mCherry::*lgg-1* after feeding (black) or 24 h of starvation (green), feeding and simultaneous treatment with 20 μM pimozide (grey), or starvation and simultaneous treatment with 5 mM 3-methyladenine (dark green) in 1 day old adults. **b** Representative pictures of fed and starved P_*nhx-2*_::mCherry::*lgg-1* at day 1 under white light and fluorescence light. **c** Number of puncta per individual worm in P_*nhx-2*_::mCherry::*lgg-1* at different ages, fed (black) or after 24 h starvation (green). **d** Homocysteine levels in wt (black), *anmt-1*^OEx^ (grey) and *anmt-1(gk457)* (white) of mixed ages populations. **e** Homocysteine levels in wt and *anmt-1*^dopa^ (red) of mixed ages populations. **f** SAM concentration in nmol/mg protein of a mixed population of wt and *sams-1(ok3033)* (grey). **g** Number of puncta per individual worm in wt and *sams-1(ok3033)* at day 1 of adulthood after feeding or 24 h of starvation. **h** Number of puncta per individual worm in *anmt-1(gk457)*, *anmt-1(gk457);sams-1(ok3033)*-heterozygous (hets) and *anmt-1(gk457);sams-1(ok3033)* at day 1 of adulthood after feeding or 24 h of starvation. **i** Number of puncta per individual worm in *anmt-1*^OEx^, *anmt-1*^OEx^*;sams-1(ok3033)*-heterozygous (hets) and *anmt-1*^OEx^*;sams-1(ok3033)* at day 1 of adulthood after feeding or 24 h of starvation. **j** Presence of CEP, ADE, and PDE cell bodies in *sams-1(ok3033)* (grey) and *anmt-1*^dopa^*;sams-1(ok3033)* (dark blue) compared to wt at day 15 of adulthood. *: p < 0.05, **: p < 0.01, ***: p < 0.001.
(PDF)

S6 Fig ANMT-1 regulates autophagy via the NPRL-2 pathway. **a** Presence of CEP, ADE, and PDE cell bodies in *anmt-1*^dopa^ with a neuronal RNAi-sensitive background treated with RNAi against *lcmt-1* (blue) and *nprl-2* (red) compared to control RNAi (EV; black) and wt EV (patterned) at day 15 of adulthood. **b** Presence of CEP, ADE, and PDE cell bodies in *anmt-1*^dopa^ with a neuronal RNAi-sensitive background treated with RNAi against *lcmt-1* and *nprl-2* compared to EV and wt EV at day 5 of adulthood. **c and d** Presence of CEP, ADE, and PDE cell bodies in wt with a neuronal RNAi-sensitive background treated with RNAi against *lcmt-1* and *nprl-2* compared to EV at **c** day 5 and **d** day 15 of adulthood. **e** DA neuronal morphology in *anmt-1*^dopa^ with a neuronal RNAi-sensitive background treated with RNAi against *lcmt-1* and *nprl-2* compared to EV and wt EV at day 5 of adulthood. **f and g** DA neuronal morphology in wt with a neuronal RNAi-sensitive background treated with RNAi against *lcmt-1* and *nprl-2* compared to EV at **f** day 5 and **g** day 15 of adulthood. **h** Lifespan of wt with a neuronal RNAi-sensitive background treated with RNAi against *lcmt-1* and *nprl-2* compared to EV. **i** Number of puncta per individual wt worm at day 5 treated with RNAi against *lcmt-1* and *nprl-2* compared to EV after feeding (black) or 24 h of starvation (green). **j** Number of puncta per individual *anmt-1*^OEx^ worm at day 1 treated with RNAi against *lcmt-1* and *nprl-2* compared to wt EV. *: p < 0.05, **: p < 0.01, ***: p < 0.001.
(PDF)

S7 Fig *anmt-1*^dopa^ rescues DA neurodegeneration in toxin-induced and genetic *C*. *elegans* models of PD. **a** DA neuronal morphology of wt and *anmt-1*^dopa^ categorized in no (white), slight (light grey), and severe neurodegeneration (dark grey) treated with β-hexachlorocyclohexane (β-HCH; 1 mM) compared to DMSO, paraquat (PQ; 300 μM) and 6-hydroxydopamine (6-OHDA; 1mM) compared to water at L4. **b** Representative pictures of wt and *anmt-1*^dopa^ with treatments from **a** at L4. **c** DA neuronal morphology of wt and *anmt-1*^dopa^ categorized in no, slight, and severe neurodegeneration treated with β-HCH (1 mM) compared to DMSO, PQ (300 μM) and 6-OHDA (1mM) compared to water at 10 days of adulthood. See S1 Table for statistics. **d** Representative pictures of wt and *anmt-1*^dopa^ with treatments from **c** at day 5. e Representive pictures of wt and *anmt-1*^dopa^ with treatments from **7a** at day 10. *: p < 0.05, **: p < 0.01, ***: p < 0.001.
(PDF)

S8 Fig *anmt-1*^dopa^ rescues DA neurodegeneration in toxin-induced and genetic *C*. *elegans* models of PD. **a** Presence of CEP, ADE, and PDE cell bodies in *anmt-1*^dopa^ (grey), *SNCA-*A53T^dopa^ (dark green), *anmt-1*^dopa^;*SNCA-*A53T^dopa^ (green), *pdr-1(gk488)* (purple) and *anmt-1*^dopa^;*pdr-1(gk488);* (light purple) compared to wt (black) at day 5. **b** Abnormal DA cell body positioning at day 5 of adulthood. **c** CEP dendrite dysmorphia at day 5. **d** DA neuronal axonal breaks at day 5 of adulthood. **e** Presence of CEP, ADE, and PDE cell bodies in *anmt-1*^dopa^, *SNCA-*A53T^dopa^, *anmt-1*^dopa^;*SNCA-*A53T^dopa^, *pdr-1(gk488)* and *anmt-1*^dopa^;*pdr-1(gk488)* compared to wt at day 15 of adulthood. **f** CEP dendrite dysmorphia at day 15 of adulthood. **g** DA neuronal axonal breaks at day 15 of adulthood. *: p < 0.05, **: p < 0.01, ***: p < 0.001.
(PDF)

S1 Table Statistics for toxin induced DA neurodegeneration in wt and *anmt-1*^dopa^, Fig 7A, 7B and 7C. Significant values are shaded in grey.
(DOCX)
