# Supplementary figures and images for: Nicotinamide-N-methyltransferase controls behavior, neurodegeneration and lifespan by regulating neuronal autophagy
Source: PLoS Genet. 2018 Sep 7;14(9):e1007561. doi: 10.1371/journal.pgen.1007561 (PMC6191153; doi:10.1371/journal.pgen.1007561)

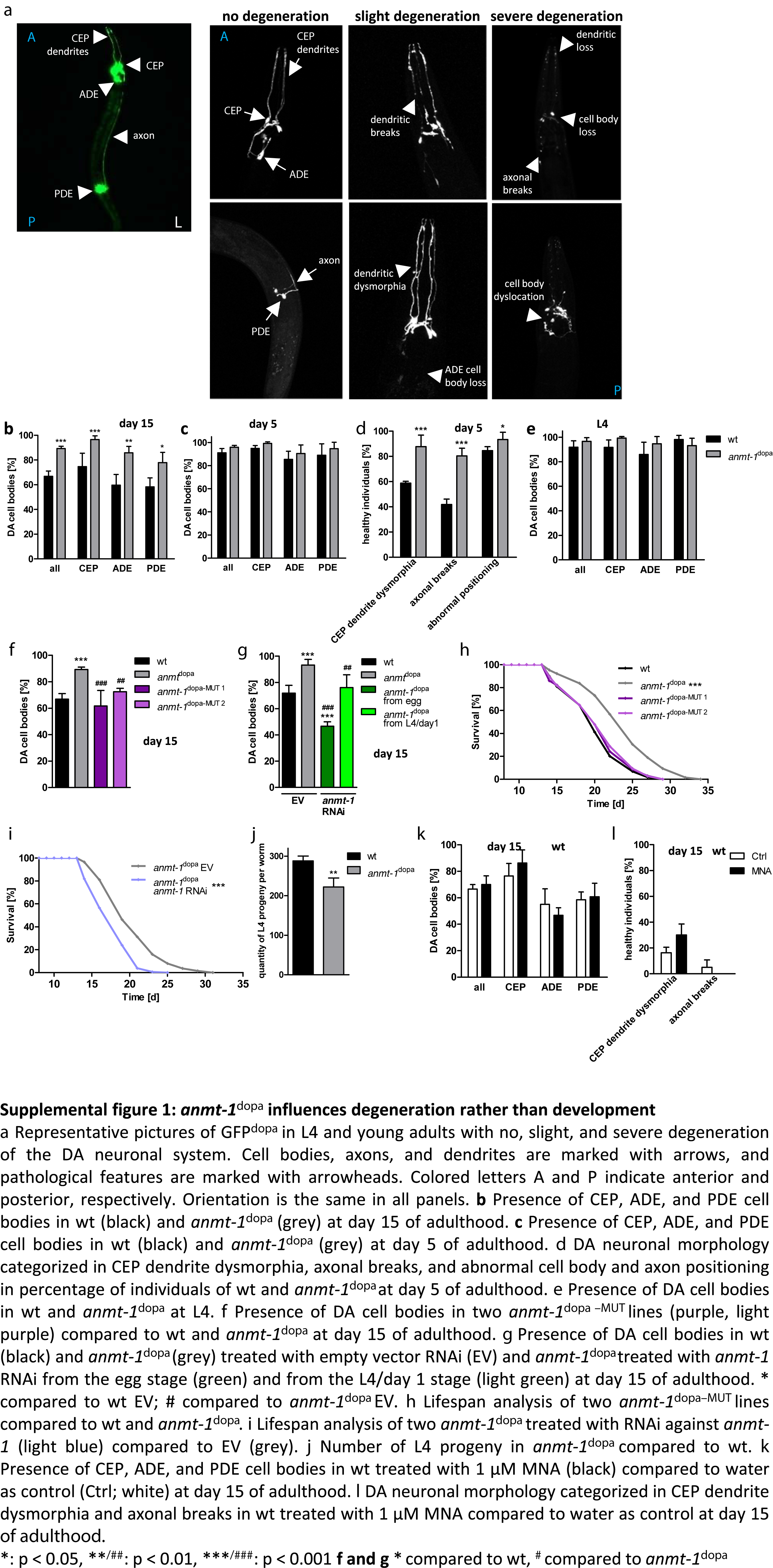

Supplement: S1 Fig — a Representative pictures of GFPdopa in L4 and young adults with no, slight, and severe degeneration of the DA neuronal system. Cell bodies, axons, and dendrites are marked with arrows, and pathological features are marked with arrowheads. Colored letters A and P indicate anterior and posterior, respectively. Orientation is the same in all panels. b Presence of CEP, ADE, and PDE cell bodies in wt (black) and anmt-1dopa (grey) at day 15 of adulthood. c Presence of CEP, ADE, and PDE cell bodies in wt (black) and anmt-1dopa (grey) at day 5 of adulthood. d DA neuronal morphology categorized in CEP dendrite dysmorphia, axonal breaks, and abnormal cell body and axon positioning in percentage of individuals of wt and anmt-1dopa at day 5 of adulthood. e Presence of DA cell bodies in wt and anmt-1dopa at L4. f Presence of DA cell bodies in two anmt-1dopa–MUT lines (purple, light purple) compared to wt and anmt-1dopa at day 15 of adulthood. g Presence of DA cell bodies in wt (black) and anmt-1dopa (grey) treated with empty vector RNAi (EV) and anmt-1dopa treated with anmt-1 RNAi from the egg stage (green) and from the L4/day 1 stage (light green) at day 15 of adulthood. * compared to wt EV; # compared to anmt-1dopa EV. h Lifespan analysis of two anmt-1dopa–MUT lines compared to wt and anmt-1dopa. i Lifespan analysis of two anmt-1dopa treated with RNAi against anmt-1 (light blue) compared to EV (grey). j Number of L4 progeny in anmt-1dopa compared to wt. k Presence of CEP, ADE, and PDE cell bodies in wt treated with 1 μM MNA (black) compared to water as control (Ctrl; white) at day 15 of adulthood. l DA neuronal morphology categorized in CEP dendrite dysmorphia and axonal breaks in wt treated with 1 μM MNA compared to water as control at day 15 of adulthood. *: p < 0.05, **/##: p < 0.01, ***/###: p < 0.001 f and g * compared to wt, # compared to anmt-1dopa (TIF) [file pgen.1007561.s001.tif]
