# Supplementary material for: Nicotinamide-N-methyltransferase controls behavior, neurodegeneration and lifespan by regulating neuronal autophagy
Source: PLoS Genet. 2018 Sep 7;14(9):e1007561. doi: 10.1371/journal.pgen.1007561 (PMC6191153; doi:10.1371/journal.pgen.1007561)
Supplement: S2 Fig — a Representative pictures of L4 anmt-1GABA marked with mCherryGABA. GABAergic neuronal cell bodies are marked with arrows, and commissures and axons are marked with arrowheads. b GABAergic cell bodies in wt (black) and anmt-1GABA (grey) at day 15 of adulthood. c GABAergic commissures in wt and anmt-1GABA at day 15 of adulthood. d Presence of GABAergic axonal breaks in wt and anmt-1GABA at day 15 of adulthood in percentage of healthy individuals. e Lifespan analysis of anmt-1GABA compared to wt. f Number of L4 progeny in anmt-1GABA compared to wt. g Locomotion over a period of 10 h of anmt-1GABA compared to wt at day 5 of adulthood. h Locomotion over a period of 10 h of anmt-1GABA compared to wt at day 10 of adulthood. i GABA concentration in nmol/mg protein of a mixed population of wt and anmt-1GABA. ***: p < 0.001. (PDF) [file pgen.1007561.s002.pdf]

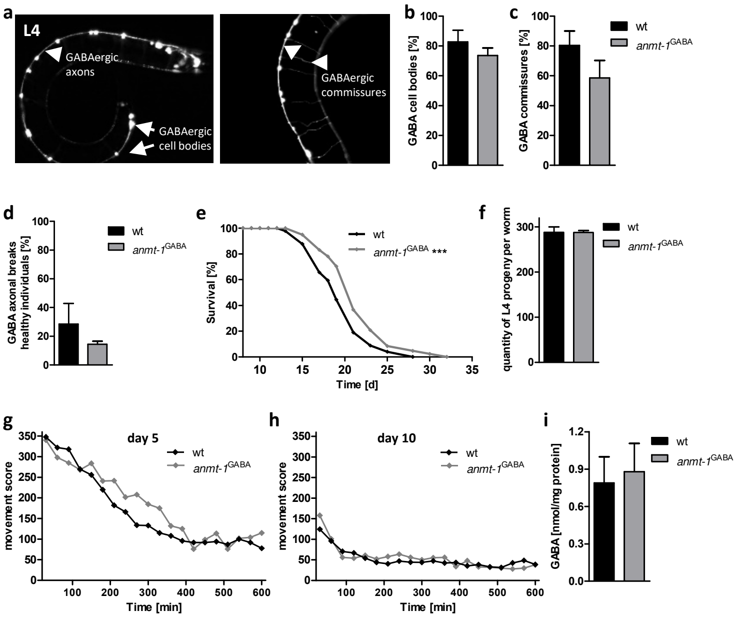

Supplemental figure 2: *anmt-1* expression in the GABAergic neuronal system shows less effects than in the DA neuronal system

a Representative pictures of L4 *anmt-1*^{GABA} marked with mCherry^{GABA}. GABAergic neuronal cell bodies are marked with arrows, and commissures and axons are marked with arrowheads. **b** GABAergic cell bodies in wt (black) and *anmt-1*^{GABA} (grey) at day 15 of adulthood. **c** GABAergic commissures in wt and *anmt-1*^{GABA} at day 15 of adulthood. **d** Presence of GABAergic axonal breaks in wt and *anmt-1*^{GABA} at day 15 of adulthood in percentage of healthy individuals. **e** Lifespan analysis of *anmt-1*^{GABA} compared to wt. **f** Number of L4 progeny in *anmt-1*^{GABA} compared to wt. **g** Locomotion over a period of 10 h of *anmt-1*^{GABA} compared to wt at day 5 of adulthood. **h** Locomotion over a period of 10 h of *anmt-1*^{GABA} compared to wt at day 10 of adulthood. **i** GABA concentration in nmol/mg protein of a mixed population of wt and *anmt-1*^{GABA}.

***: $p < 0.001$
