# Supplementary material for: Nicotinamide-N-methyltransferase controls behavior, neurodegeneration and lifespan by regulating neuronal autophagy
Source: PLoS Genet. 2018 Sep 7;14(9):e1007561. doi: 10.1371/journal.pgen.1007561 (PMC6191153; doi:10.1371/journal.pgen.1007561)
Supplement: S3 Fig — a–c Basal slowing response tested as quantity of body bends per animal per 20 sec in wt and anmt-1dopa animals on plates with (black) or without bacteria (white) at a day 1, b day 5, and c day 10 of adulthood. d Quantity of body bends per animal per 20 sec in two anmt-1dopa-MUT lines on plates with or without bacteria at day 5 of adulthood. e and f Dopamine-dependent behaviors in wt, wt after 4–6 hours 50 mM dopamine pre-treatment (wt + dopamine; grey), anmt-1dopa, and anmt-1dopa after 4–6 hours 50 mM dopamine pre-treatment (anmt-1dopa + dopamine; rose) at day 5 of adulthood. e Basal slowing index. f Chemotaxis index. g Lifespan of anmt-1dopa;cat-2(n4547) (grey) compared to cat-2(n4547) (black). h Lifespan of dop-3(vs106);anmt-1dopa (grey) compared to dop-3(vs106) (black). i Presence of CEP, ADE, and PDE cell bodies in cat-2(n4547);anmt-1dopa (light turquoise) compared to cat-2(n4547) (turquoise), and dop-3(vs106);anmt-1dopa (light pink) compared to dop-3(vs106) (pink) at day 15 of adulthood. *: p < 0.05, ** and ##: p < 0.01, ***: p < 0.001, e * compared to cat-2(n4547), # compared to dop-3(vs106). (PDF) [file pgen.1007561.s003.pdf]

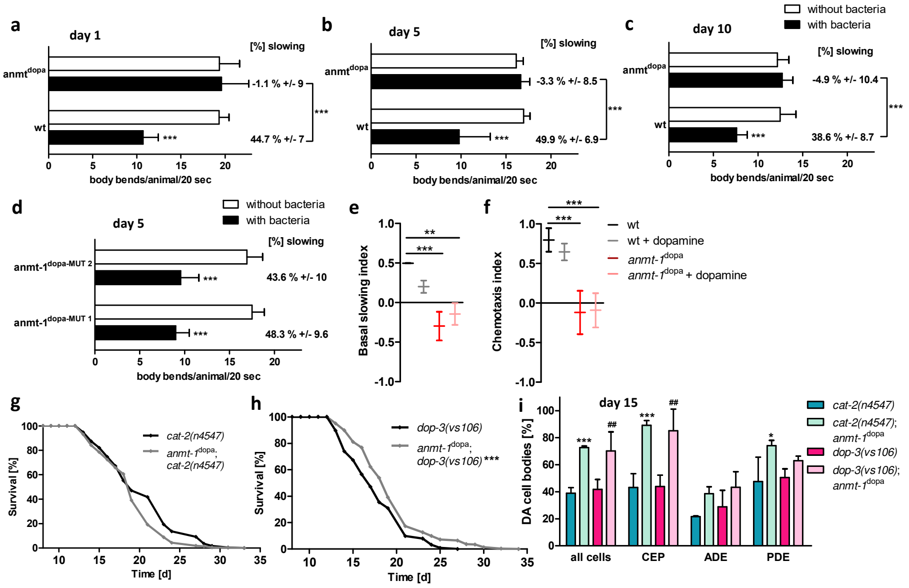

Supplemental figure 3: Neuronal autophagy mediates *anmt-1*^{dopa} induced phenotypes

a – c Basal slowing response tested as quantity of body bends per animal per 20 sec in wt and *anmt-1*^{dopa} animals on plates with (black) or without bacteria (white) at **a** day 1, **b** day 5, and **c** day 10 of adulthood. **d** Quantity of body bends per animal per 20 sec in two *anmt-1*$^{dopa\text{-}MUT}$ lines on plates with or without bacteria at day 5 of adulthood. **e and f** Dopamine-dependent behaviors in wt, wt after 4 – 6 hours 50 mM dopamine pre-treatment (wt + dopamine; grey), *anmt-1*^{dopa}, and *anmt-1*^{dopa} after 4 – 6 hours 50 mM dopamine pre-treatment (*anmt-1*^{dopa} + dopamine; rose) at day 5 of adulthood. **e** Basal slowing index. **f** Chemotaxis index. **g** Lifespan of *anmt-1*^{dopa};*cat-2(n4547)* (grey) compared to *cat-2(n4547)* (black). **h** Lifespan of *dop-3(vs106)*;*anmt-1*^{dopa} (grey) compared to *dop-3(vs106)* (black). **i** Presence of CEP, ADE, and PDE cell bodies in *cat-2(n4547);anmt-1*^{dopa} (light turquoise) compared to *cat-2(n4547)* (turquoise), and *dop-3(vs106)*;*anmt-1*^{dopa} (light pink) compared to *dop-3(vs106)* (pink) at day 15 of adulthood.

*: $p < 0.05$, ** and $^{\#\#}$: $p < 0.01$, ***: $p < 0.001$, **e** * compared to *cat-2(n4547)*, $^{\#}$ compared to *dop-3(vs106).*
