# Supplementary material for: Nicotinamide-N-methyltransferase controls behavior, neurodegeneration and lifespan by regulating neuronal autophagy
Source: PLoS Genet. 2018 Sep 7;14(9):e1007561. doi: 10.1371/journal.pgen.1007561 (PMC6191153; doi:10.1371/journal.pgen.1007561)
Supplement: S4 Fig — a Presence of CEP, ADE, and PDE cell bodies in anmt-1dopa with a neuronal RNAi-sensitive background treated with RNAi against bec-1 (orange), atg-13 (green), or lgg-1 (purple) compared to control RNAi (EV; black) and wt EV (patterned) at day 15 of adulthood. b Basal slowing index of wt treated with RNAi against bec-1, atg-13 or lgg-1 compared to EV. c Presence of CEP, ADE, and PDE cell bodies in wt with a neuronal RNAi-sensitive background treated with RNAi against bec-1, atg-13, or lgg-1 compared to EV at day 15 of adulthood. d DA neuronal morphology in wt with a neuronal RNAi-sensitive background treated with RNAi against bec-1, atg-13, or lgg-1 compared to EV at day 15 of adulthood. e Presence of CEP, ADE, and PDE cell bodies in anmt-1dopa with a neuronal RNAi-sensitive background treated with RNAi against bec-1, atg-13, or lgg-1 compared to EV and wt EV at day 5 of adulthood. f DA neuronal morphology in anmt-1dopa with a neuronal RNAi-sensitive background treated with RNAi against bec-1, atg-13, or lgg-1 compared to EV and wt EV at day 5 of adulthood. g DA neuronal morphology in wt with a neuronal RNAi-sensitive background treated with RNAi against bec-1, atg-13, or lgg-1 compared to EV at day 5. h DA neuronal morphology in wt with a neuronal RNAi-sensitive background treated with RNAi against bec-1, atg-13, or lgg-1 compared to EV at day 5 of adulthood. i Lifespan of wt with a neuronal RNAi-sensitive background treated with RNAi against bec-1, atg-13 and lgg-1 compared to EV. *: p < 0.05, **: p < 0.01, ***: p < 0.001. (PDF) [file pgen.1007561.s004.pdf]

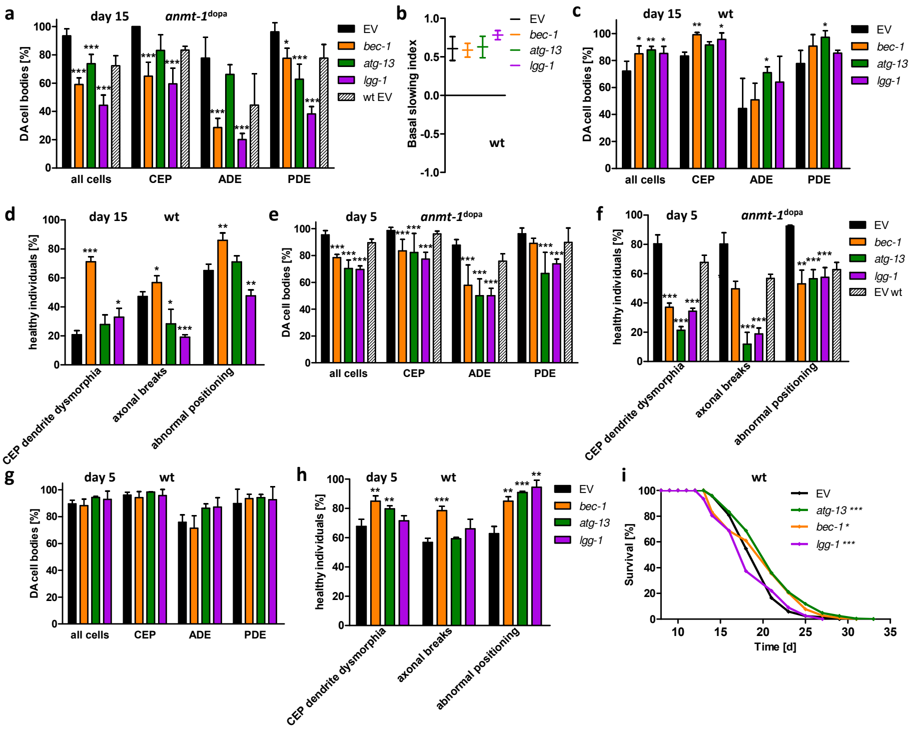

**Supplemental figure 4: Neuronal autophagy mediates *anmt-1*$^{dopa}$ induced phenotypes**

**a** Presence of CEP, ADE, and PDE cell bodies in *anmt-1*$^{dopa}$ with a neuronal RNAi-sensitive background treated with RNAi against *bec-1* (orange), *atg-13* (green), or *lgg-1* (purple) compared to control RNAi (EV; black) and wt EV (patterned) at day 15 of adulthood. **b** Basal slowing index of wt treated with RNAi against *bec-1*, *atg-13* or *lgg-1* compared to EV. **c** Presence of CEP, ADE, and PDE cell bodies in wt with a neuronal RNAi-sensitive background treated with RNAi against *bec-1*, *atg-13*, or *lgg-1* compared to EV at day 15 of adulthood. **d** DA neuronal morphology in wt with a neuronal RNAi-sensitive background treated with RNAi against *bec-1*, *atg-13*, or *lgg-1* compared to EV at day 15 of adulthood. **e** Presence of CEP, ADE, and PDE cell bodies in *anmt-1*$^{dopa}$ with a neuronal RNAi-sensitive background treated with RNAi against *bec-1*, *atg-13*, or *lgg-1* compared to EV and wt EV at day 5 of adulthood. **f** DA neuronal morphology in *anmt-1*$^{dopa}$ with a neuronal RNAi-sensitive background treated with RNAi against *bec-1*, *atg-13*, or *lgg-1* compared to EV and wt EV at day 5 of adulthood. **g** DA neuronal morphology in wt with a neuronal RNAi-sensitive background treated with RNAi against *bec-1*, *atg-13*, or *lgg-1* compared to EV at day 5. **h** DA neuronal morphology in wt with a neuronal RNAi-sensitive background treated with RNAi against *bec-1*, *atg-13*, or *lgg-1* compared to EV at day 5 of adulthood. **i** Lifespan of wt with a neuronal RNAi-sensitive background treated with RNAi against *bec-1*, *atg-13* and *lgg-1* compared to EV.

*: $p < 0.05$, **: $p < 0.01$, ***: $p < 0.001$
