# Supplementary material for: Nicotinamide-N-methyltransferase controls behavior, neurodegeneration and lifespan by regulating neuronal autophagy
Source: PLoS Genet. 2018 Sep 7;14(9):e1007561. doi: 10.1371/journal.pgen.1007561 (PMC6191153; doi:10.1371/journal.pgen.1007561)
Supplement: S5 Fig — a Number of puncta per individual worm in Pnhx-2::mCherry::lgg-1 after feeding (black) or 24 h of starvation (green), feeding and simultaneous treatment with 20 μM pimozide (grey), or starvation and simultaneous treatment with 5 mM 3-methyladenine (dark green) in 1 day old adults. b Representative pictures of fed and starved Pnhx-2::mCherry::lgg-1 at day 1 under white light and fluorescence light. c Number of puncta per individual worm in Pnhx-2::mCherry::lgg-1 at different ages, fed (black) or after 24 h starvation (green). d Homocysteine levels in wt (black), anmt-1OEx (grey) and anmt-1(gk457) (white) of mixed ages populations. e Homocysteine levels in wt and anmt-1dopa (red) of mixed ages populations. f SAM concentration in nmol/mg protein of a mixed population of wt and sams-1(ok3033) (grey). g Number of puncta per individual worm in wt and sams-1(ok3033) at day 1 of adulthood after feeding or 24 h of starvation. h Number of puncta per individual worm in anmt-1(gk457), anmt-1(gk457);sams-1(ok3033)-heterozygous (hets) and anmt-1(gk457);sams-1(ok3033) at day 1 of adulthood after feeding or 24 h of starvation. i Number of puncta per individual worm in anmt-1OEx, anmt-1OEx;sams-1(ok3033)-heterozygous (hets) and anmt-1OEx;sams-1(ok3033) at day 1 of adulthood after feeding or 24 h of starvation. j Presence of CEP, ADE, and PDE cell bodies in sams-1(ok3033) (grey) and anmt-1dopa;sams-1(ok3033) (dark blue) compared to wt at day 15 of adulthood. *: p < 0.05, **: p < 0.01, ***: p < 0.001. (PDF) [file pgen.1007561.s005.pdf]

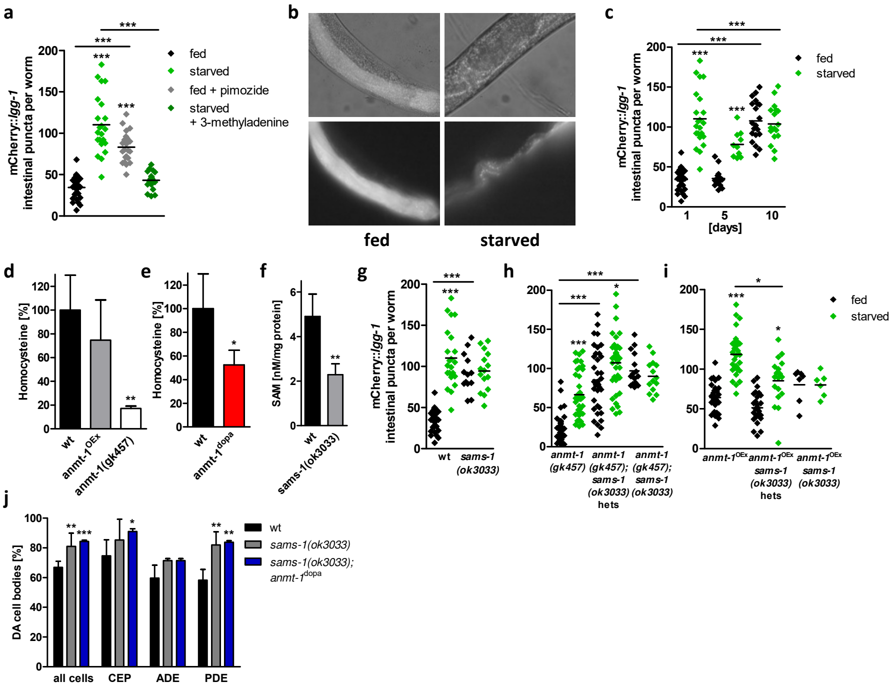

Supplemental figure 5: ANMT-1 regulates autophagy by controlling SAM levels

a Number of puncta per individual worm in P_{nhx-2}::mCherry::*lgg-1* after feeding (black) or 24 h of starvation (green), feeding and simultaneous treatment with 20 µM pimozide (grey), or starvation and simultaneous treatment with 5 mM 3-methyladenine (dark green) in 1 day old adults. **b** Representative pictures of fed and starved P_{nhx-2}::mCherry::*lgg-1* at day 1 under white light and fluorescence light. **c** Number of puncta per individual worm in P_{nhx-2}::mCherry::*lgg-1* at different ages, fed (black) or after 24 h starvation (green). **d** Homocysteine levels in wt (black), *anmt-1*^{OEx} (grey) and *anmt-1(gk457)* (white) of mixed ages populations. **e** Homocysteine levels in wt and *anmt-1*^{dopa} (red) of mixed ages populations. **f** SAM concentration in nmol/mg protein of a mixed population of wt and *sams-1(ok3033)* (grey). **g** Number of puncta per individual worm in wt and *sams-1(ok3033)* at day 1 of adulthood after feeding or 24 h of starvation. **h** Number of puncta per individual worm in *anmt-1(gk457), anmt-1(gk457);sams-1(ok3033)*-heterozygous (hets) and *anmt-1(gk457);sams-1(ok3033)* at day 1 of adulthood after feeding or 24 h of starvation. **i** Number of puncta per individual worm in *anmt-1*^{OEx}, *anmt-1*^{OEx}*;sams-1(ok3033)*-heterozygous (hets) and *anmt-1*^{OEx}*;sams-1(ok3033)* at day 1 of adulthood after feeding or 24 h of starvation. **j** Presence of CEP, ADE, and PDE cell bodies in *sams-1(ok3033)* (grey) and *anmt-1*^{dopa}*;sams-1(ok3033)* (dark blue) compared to wt at day 15 of adulthood.

*: $p < 0.05$, **: $p < 0.01$, ***: $p < 0.001$
