# Supplementary material for: Nicotinamide-N-methyltransferase controls behavior, neurodegeneration and lifespan by regulating neuronal autophagy
Source: PLoS Genet. 2018 Sep 7;14(9):e1007561. doi: 10.1371/journal.pgen.1007561 (PMC6191153; doi:10.1371/journal.pgen.1007561)
Supplement: S6 Fig — a Presence of CEP, ADE, and PDE cell bodies in anmt-1dopa with a neuronal RNAi-sensitive background treated with RNAi against lcmt-1 (blue) and nprl-2 (red) compared to control RNAi (EV; black) and wt EV (patterned) at day 15 of adulthood. b Presence of CEP, ADE, and PDE cell bodies in anmt-1dopa with a neuronal RNAi-sensitive background treated with RNAi against lcmt-1 and nprl-2 compared to EV and wt EV at day 5 of adulthood. c and d Presence of CEP, ADE, and PDE cell bodies in wt with a neuronal RNAi-sensitive background treated with RNAi against lcmt-1 and nprl-2 compared to EV at c day 5 and d day 15 of adulthood. e DA neuronal morphology in anmt-1dopa with a neuronal RNAi-sensitive background treated with RNAi against lcmt-1 and nprl-2 compared to EV and wt EV at day 5 of adulthood. f and g DA neuronal morphology in wt with a neuronal RNAi-sensitive background treated with RNAi against lcmt-1 and nprl-2 compared to EV at f day 5 and g day 15 of adulthood. h Lifespan of wt with a neuronal RNAi-sensitive background treated with RNAi against lcmt-1 and nprl-2 compared to EV. i Number of puncta per individual wt worm at day 5 treated with RNAi against lcmt-1 and nprl-2 compared to EV after feeding (black) or 24 h of starvation (green). j Number of puncta per individual anmt-1OEx worm at day 1 treated with RNAi against lcmt-1 and nprl-2 compared to wt EV. *: p < 0.05, **: p < 0.01, ***: p < 0.001. (PDF) [file pgen.1007561.s006.pdf]

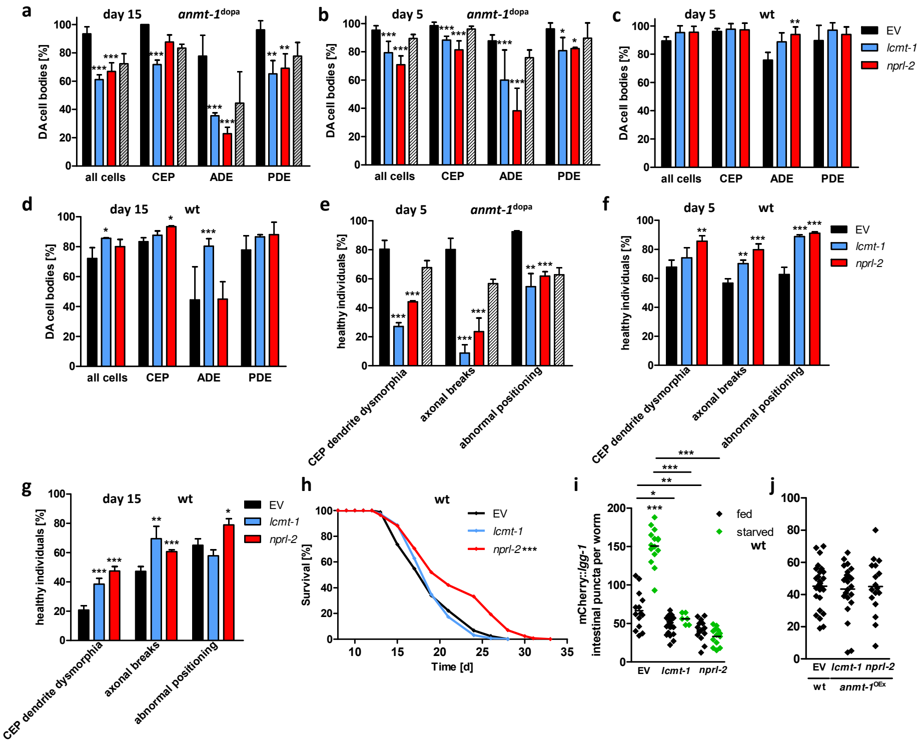

Supplemental figure 6: ANMT-1 regulates autophagy via the NPRL-2 pathway

a Presence of CEP, ADE, and PDE cell bodies in *anmt-1*^{dopa} with a neuronal RNAi-sensitive background treated with RNAi against *lcmt-1* (blue) and *nprl-2* (red) compared to control RNAi (EV; black) and wt EV (patterned) at day 15 of adulthood. **b** Presence of CEP, ADE, and PDE cell bodies in *anmt-1*^{dopa} with a neuronal RNAi-sensitive background treated with RNAi against *lcmt-1* and *nprl-2* compared to EV and wt EV at day 5 of adulthood. **c and d** Presence of CEP, ADE, and PDE cell bodies in wt with a neuronal RNAi-sensitive background treated with RNAi against *lcmt-1* and *nprl-2* compared to EV at **c** day 5 and **d** day 15 of adulthood. **e** DA neuronal morphology in *anmt-1*^{dopa} with a neuronal RNAi-sensitive background treated with RNAi against *lcmt-1* and *nprl-2* compared to EV and wt EV at day 5 of adulthood. **f and g** DA neuronal morphology in wt with a neuronal RNAi-sensitive background treated with RNAi against *lcmt-1* and *nprl-2* compared to EV at **f** day 5 and **g** day 15 of adulthood. **h** Lifespan of wt with a neuronal RNAi-sensitive background treated with RNAi against *lcmt-1* and *nprl-2* compared to EV. **i** Number of puncta per individual wt worm at day 5 treated with RNAi against *lcmt-1* and *nprl-2* compared to EV after feeding (black) or 24 h of starvation (green). **j** Number of puncta per individual *anmt-1*^{OEx} worm at day 1 treated with RNAi against *lcmt-1* and *nprl-2* compared to wt EV.

*: $p < 0.05$, **: $p < 0.01$, ***: $p < 0.001$
