# Supplementary material for: Nicotinamide-N-methyltransferase controls behavior, neurodegeneration and lifespan by regulating neuronal autophagy
Source: PLoS Genet. 2018 Sep 7;14(9):e1007561. doi: 10.1371/journal.pgen.1007561 (PMC6191153; doi:10.1371/journal.pgen.1007561)
Supplement: S7 Fig — a DA neuronal morphology of wt and anmt-1dopa categorized in no (white), slight (light grey), and severe neurodegeneration (dark grey) treated with β-hexachlorocyclohexane (β-HCH; 1 mM) compared to DMSO, paraquat (PQ; 300 μM) and 6-hydroxydopamine (6-OHDA; 1mM) compared to water at L4. b Representative pictures of wt and anmt-1dopa with treatments from a at L4. c DA neuronal morphology of wt and anmt-1dopa categorized in no, slight, and severe neurodegeneration treated with β-HCH (1 mM) compared to DMSO, PQ (300 μM) and 6-OHDA (1mM) compared to water at 10 days of adulthood. See S1 Table for statistics. d Representative pictures of wt and anmt-1dopa with treatments from c at day 5. e Representive pictures of wt and anmt-1dopa with treatments from 7a at day 10. *: p < 0.05, **: p < 0.01, ***: p < 0.001. (PDF) [file pgen.1007561.s007.pdf]

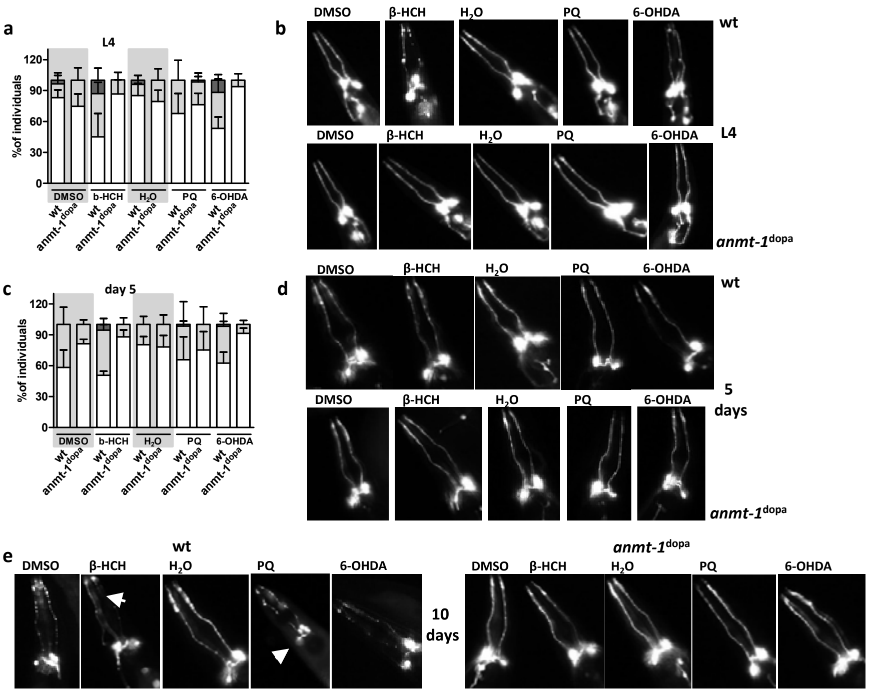

Supplemental figure 7: *anmt-1*^{dopa} rescues DA neurodegeneration in toxin-induced and genetic *C. elegans* models of PD

a DA neuronal morphology of wt and *anmt-1*^{dopa} categorized in no (white), slight (light grey), and severe neurodegeneration (dark grey) treated with β-hexachlorocyclohexane (β-HCH; 1 mM) compared to DMSO, paraquat (PQ; 300 µM) and 6-hydroxydopamine (6-OHDA; 1mM) compared to water at L4. **b** Representative pictures of wt and *anmt-1*^{dopa} with treatments from **a** at L4. **c** DA neuronal morphology of wt and *anmt-1*^{dopa} categorized in no, slight, and severe neurodegeneration treated with β-HCH (1 mM) compared to DMSO, PQ (300 µM) and 6-OHDA (1mM) compared to water at 10 days of adulthood. See tabl. S1 for statistics. **d** Representative pictures of wt and *anmt-1*^{dopa} with treatments from **c** at day 5. **e** Representative pictures of wt and *anmt-1*^{dopa} with treatments from **7a** at day 10.

*: $p < 0.05$, **: $p < 0.01$, ***: $p < 0.001$
