# Supplementary material for: Nicotinamide-N-methyltransferase controls behavior, neurodegeneration and lifespan by regulating neuronal autophagy
Source: PLoS Genet. 2018 Sep 7;14(9):e1007561. doi: 10.1371/journal.pgen.1007561 (PMC6191153; doi:10.1371/journal.pgen.1007561)
Supplement: S8 Fig — a Presence of CEP, ADE, and PDE cell bodies in anmt-1dopa (grey), SNCA-A53Tdopa (dark green), anmt-1dopa;SNCA-A53Tdopa (green), pdr-1(gk488) (purple) and anmt-1dopa;pdr-1(gk488); (light purple) compared to wt (black) at day 5. b Abnormal DA cell body positioning at day 5 of adulthood. c CEP dendrite dysmorphia at day 5. d DA neuronal axonal breaks at day 5 of adulthood. e Presence of CEP, ADE, and PDE cell bodies in anmt-1dopa, SNCA-A53Tdopa, anmt-1dopa;SNCA-A53Tdopa, pdr-1(gk488) and anmt-1dopa;pdr-1(gk488) compared to wt at day 15 of adulthood. f CEP dendrite dysmorphia at day 15 of adulthood. g DA neuronal axonal breaks at day 15 of adulthood. *: p < 0.05, **: p < 0.01, ***: p < 0.001. (PDF) [file pgen.1007561.s008.pdf]

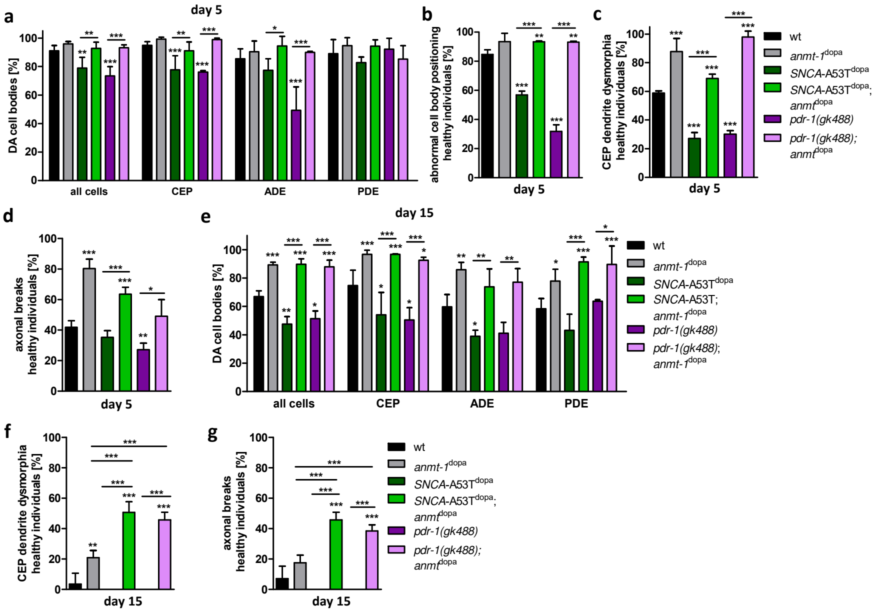

Supplemental figure 8: *anmt-1*^{dopa} rescues DA neurodegeneration in toxin-induced and genetic *C. elegans* models of PD

a Presence of CEP, ADE, and PDE cell bodies in *anmt-1*^{dopa} (grey), *SNCA*-A53T^{dopa} (dark green), *anmt-1*^{dopa};*SNCA*-A53T^{dopa} (green), *pdr-1(gk488)* (purple) and *anmt-1*^{dopa};*pdr-1(gk488);* (light purple) compared to wt (black) at day 5. **b** Abnormal DA cell body positioning at day 5 of adulthood. **c** CEP dendrite dysmorphia at day 5. **d** DA neuronal axonal breaks at day 5 of adulthood. **e** Presence of CEP, ADE, and PDE cell bodies in *anmt-1*^{dopa}, *SNCA*-A53T^{dopa}, *anmt-1*^{dopa};*SNCA*-A53T^{dopa}, *pdr-1(gk488)* and *anmt-1*^{dopa};*pdr-1(gk488)* compared to wt at day 15 of adulthood. **f** CEP dendrite dysmorphia at day 15 of adulthood. **g** DA neuronal axonal breaks at day 15 of adulthood.

*: $p < 0.05$, **: $p < 0.01$, ***: $p < 0.001$
