# Supplementary material for: Nicotinamide-N-methyltransferase controls behavior, neurodegeneration and lifespan by regulating neuronal autophagy
Source: PLoS Genet. 2018 Sep 7;14(9):e1007561. doi: 10.1371/journal.pgen.1007561 (PMC6191153; doi:10.1371/journal.pgen.1007561)
Supplement: S1 Table — Significant values are shaded in grey. (DOCX) [file pgen.1007561.s009.docx]

**Supplementary table 1:** Statistics for toxin induced DA neurodegeneration in wt and *anmt-1*^dopa^ from figure 7 and S7

|  | **toxins** | **wt** | | | ***anmt-1*^dopa^** | | | **wt vs. *anmt-1*^dopa^** | | |
| --- | --- | --- | --- | --- | --- | --- | --- | --- | --- | --- |
|  |  | **none** | **slight** | **severe** | **none** | **slight** | **severe** | **none** | **slight** | **severe** |
| **L4** | DMSO vs. β-HCH | 0,0190 | 0,0062 | 0,2194 | 0,1997 | 0,1438 |  | 0,0305 | 0,0127 | 0,1219 |
|  | H_2_O vs. PQ | 0,1803 | 0,0931 | 0,1386 | 0,6988 | 0,8522 | 0,3559 | 0,4716 | 0,3682 | 0,3559 |
|  | H_2_O vs. 6-OHDA | 0,0156 | 0,0340 | 0,0991 | 0,1016 | 0,0619 |  | 0,0052 | 0,0103 | 0,0199 |
| **5 days** | DMSO vs. β-HCH | 0,4896 | 0,8543 | 0,1074 | 0,1098 | 0,1659 |  | 0,0011 | 0,0029 | 0,1765 |
|  | H_2_O vs. PQ | 0,2560 | 0,3362 | 0,3559 | 0,7496 | 0,7733 |  | 0,5297 | 0,6177 |  |
|  | H_2_O vs. 6-OHDA | 0,0480 | 0,0844 | 0,2856 | 0,064 | 0,093 |  | 0,0099 | 0,0198 | 0,4950 |
| **10 days** | DMSO vs. β-HCH | 0,2938 | 0,4650 | 0,0855 | 0,3992 | 0,4815 |  | 0,0002 | 0,0011 | 0,0010 |
|  | H_2_O vs. PQ | 0,0030 | 0,0257 | 0,1256 | 0,7052 | 0,5177 | 0,3910 | 0,0033 | 0,0041 | 0,1810 |
|  | H_2_O vs. 6-OHDA | 0,0017 | 0,0041 | 0,0732 | 0,2974 | 0,8411 | 0,5761 | 0,0005 | 0,0057 | 0,0328 |

Significant values are shaded in grey.
